# Pro- and anti-inflammatory cytokines and osteoclastogenesis-related factors in peri-implant diseases: systematic review and meta-analysis

**DOI:** 10.1186/s12903-023-03072-1

**Published:** 2023-06-24

**Authors:** Jovânia Alves Oliveira, Roberta de Oliveira Alves, Isabella Mazarelo Nascimento, Marco Antonio Rimachi Hidalgo, Raquel Mantuaneli Scarel-Caminaga, Suzane Cristina Pigossi

**Affiliations:** 1 School of Dentistry, Alfenas Federal University (Unifal-MG), Alfenas, Minas Gerais Brazil; 2 Department of Periodontology, School of Dentistry, School of Dentistry, Federal University of Uberlândia - UFU, Umuarama Campus, Bloco UMU4L, Pará Avenue, Uberlândia, Minas Gerais 1720, 38405-320 Brazil; 3 Department of Morphology, Genetics, Orthodontics and Pediatric Dentistry, School of Dentistry at Araraquara, (FOAr/UNESP), UNESP- São Paulo State University, Araraquara, São Paulo, Brazil

**Keywords:** Peri-implantitis, Cytokines, Bone resorption, Dental implants

## Abstract

**Background:**

Pro- and anti-inflammatory cytokines are acknowledged, during inflammatory bone destruction, as key regulators of osteoclast and osteoblast differentiation and activity. However, evidence regarding the exact role of pro- and anti-inflammatory cytokines and osteoclastogenesis-related factors in peri-implant diseases is unclear. We aimed to execute a systematic review and meta-analysis about the pro- and anti-inflammatory cytokines and osteoclastogenesis-related factors levels in peri-implant diseases.

**Methods:**

The focused question was elaborated to summarize the levels of pro-and anti-inflammatory cytokines and osteoclastogenesis-related factors in tissue samples (mRNA) and biofluids (protein levels) of patients with/without peri-implant diseases. Electronic searches of the PubMed, Cochrane Controlled Trials Registry, Web of Science, EMBASE, Scopus and Google scholar databases were conducted for publications up to March 2023. Meta-analysis evaluating the mediator´s levels (protein levels by ELISA) in peri-implant crevicular fluid (PICF) were made. The effect size was estimated and reported as the mean difference. The 95% confidence interval was estimated for each mediator, and the pooled effect was determined significant if two-sided *p*-values < 0.05 were obtained.

**Results:**

Twenty-two publications were included in the systematic review (qualitative analysis), with nine of these subjected to meta-analyses (quantitative analysis). In the qualitative analysis, higher pro-inflammatory cytokines [Interleukin (IL)-1β, IL-6] and pro-osteoclastogenic mediator [Receptor Activator of Nuclear Factor-Kappa B ligand (RANKL)] levels were observed in PICF of individuals with peri-implant diseases in comparison to healthy individuals. Higher RANKL/osteoprotegerin (OPG) ratios were observed in PICF from individuals with peri-implant diseases in comparison to healthy individuals. Meta-analysis showed higher RANKL levels in diseased groups compared to controls.

**Conclusions:**

The results showed that the levels of IL-1β, IL-6, IL-10, and RANKL/OPG are not balanced in peri-implant disease, suggesting that these mediators are involved in the host osteo-immunoinflammatory response related to peri-implantitis.

**Supplementary Information:**

The online version contains supplementary material available at 10.1186/s12903-023-03072-1.

## Introduction

Dental implants have been widely used to ensure the quality of life in partially and fully edentulous patients. Prospective studies with long follow-up periods showed survival rates varying from 89.5 to 99.2% [1–3]. However, peri-implant mucositis and peri-implantitis are chronic inflammatory conditions that can reduce dental implant predictability [4]. Peri-implant mucositis is a reversible condition caused by an inflammatory process restricted to peri-implant soft tissues, while peri-implantitis exhibits a progressive supporting bone loss [5]. The general prevalence of both conditions was estimated in a meta-analysis, being 42.9% for peri-implant mucositis and 21.7% for peri-implantitis [6].

The peri-implant tissue breakdown seems to be associated with a cytokine response to bacterial products, including endotoxins and lipopolysaccharides, that results in a local immunological response at the infection tissue [7, 8]. This immune reaction to infection is adjusted by the balance between pro-and anti-inflammatory cytokines that are acknowledged, during inflammatory bone destruction, as key regulators of osteoclast and osteoblast differentiation and activity [9–11].

In this context, the production of the pro-inflammatory cytokines, such as interleukin (IL)-1β, -6, and -12, interferon-gamma and tumor necrosis factor-alpha (TNF-a), in reaction to a periodontal infection, are responsible to stimulate tissue damage by activation of collagenase and other pro-inflammatory factors [12–15]. IL-1β manages the prostaglandin E2 production associated with hard tissue breakdown induction in periodontitis [16]. Higher levels of both mediators were found in the gingival crevicular fluid of patients with periodontal disease [17, 18]. Similarly, IL-6 increase T-lymphocyte proliferation and B-lymphocyte differentiation/immunoglobulin secretion as reported by in vitro studies [19, 20]. Moreover, IL-6 also induces bone resorption by itself and in conjunction with other bone-resorbing mediators and acts synergistically with IL-1β. The levels of both proinflammatory cytokines in peri-implant crevicular fluid (PICF) were significantly higher in sites with peri-implantitis in comparison to healthy sites [8, 21].

Anti-inflammatory cytokines, such as IL-10, IL-4 and IL-1 receptor antagonist (IL1-RA), are produced to limit the inflammatory events, revealing protective functions during tissue destruction as reported by in vitro studies [22, 23]. IL-10 is produced by T-helper 2 cells (TH2), macrophages, and B cells and acts to reduce the production of the pro-inflammatory cytokines [24, 25]. Furthermore, IL-10 acts enhanced the B cell proliferation and differentiation and favored immunoglobulins production in vitro, balancing the immune response [26]. A previous study [27] showed that higher IL-10 and lower IL-1β levels in PICF are related, clinically and radiographically, to peri-implant health.

The alveolar bone loss around dental implants seems to be controlled by the interaction of the Receptor Activator of Nuclear Factor-Kappa B ligand (RANKL), also named as TNF Receptor Superfamily Member 11 (TNFRSF11), with osteoprotegerin (OPG) whose expressions are strongly controlled by immune cell-derived inflammatory cytokines and bacterial products [28]. RANKL interacts with RANK, also named as TNF Receptor Superfamily Member 11A (TNFRSF11A), and the binding of RANKL to RANK takes place in the osteoclast precursor cells, inducing osteoclast formation and activation resulting in bone resorption, therefore, RANKL is a pro-osteoclastogenic protein [29, 30]. Instead, OPG is a decoy receptor for RANKL which inhibit osteoclastogenesis [30, 31]. A RANKL/OPG ratio was associated with bone damage by inducing osteoclast formation during the inflammation process [32]. This suggests that osteoclast activity is associated with a RANKL and OPG equilibrium [28].

Current evidence suggests that a complex set of chemokine/cytokine signaling pathways are associated with inflammation and bone resorption, the hallmarks of peri-implantitis. [31]. A greater understanding of this microenvironment around dental implants may help to monitor the health state of surrounding tissues. However, evidence regarding the exact role of pro and anti-inflammatory cytokines and osteoclastogenesis-related factors in peri-implant diseases is incomplete and unclear [33]. Based on that, we aimed to execute a systematic review and meta-analysis focusing on the levels of pro-and anti-inflammatory cytokines and osteoclastogenesis-related factors in peri-implant diseases.

## Material and methods

### Protocol

The present systematic review with meta-analysis was guided by the Preferred Reporting Items for Systematic Reviews and Meta-Analyses (PRISMA) statement and a protocol was registered in PROSPERO (ID: CRD42020213627).

### Focused question

The focused question was elaborated by PECO [population (patients containing implants with peri-implant diseases); exposure (peri-implant diseases); comparator (patients containing implants without peri-implant diseases); outcome (pro- and anti-inflammatory cytokines and osteoclastogenesis-related factors levels in tissue sample or biofluids)] principles to summarize the levels of pro- and anti-inflammatory cytokines and osteoclastogenesis-related factors in patients with/without peri-implant diseases: “Do implants with peri-implant diseases have different levels of pro- and anti-inflammatory mediators, or osteoclastogenesis-related factors compared with implants without peri-implant diseases?”.

### Eligibility criteria

The original research articles were selected according to these inclusion criteria: (i) longitudinal studies and cross-sectional studies (cohort and case–control studies); (ii) describing data about pro- and anti-inflammatory mediator profiles in a tissue sample or the subsequent biofluid PICF, saliva and blood of patients with and without peri-implant diseases; (iii) studies including statistical methods and numerical values of mean and standard deviation, with the units for quantifying mediators levels; (iv) articles published only in the English language. To include studies in the systematic review and meta-analyses, they should report both related pro- and anti-inflammatory, as well as pro-and anti-osteoclastogenic factors evaluated in the same group of individuals. Studies that evaluated only one mediator were excluded. For the systematic review (qualitative analysis), studies that investigated protein levels of modulators by ELISA and Multiplex methods were considered, because they are both immunoassays (ELISA is a single plex, while the Multiplex assess multiple different proteins simultaneously). Original research articles that did not follow all the criteria defined above were eliminated from this systematic review. Moreover, letters to the editor, historical reviews, experimental studies (animal and cellular models) and unpublished articles were also eliminated.

### Outcome measures

To assess the levels of both pro-and anti-inflammatory cytokines, or bone osteoclastogenesis-related factors levels, in individuals with and without peri-implant diseases, the primary outcome measure was the pro-and anti-inflammatory modulators levels (IL-1 and IL-10, IL-6 and IL-10, IL-1 and IL-1RA or RANKL and OPG) in sample tissue (mRNA) and biofluids (protein levels) of individuals with peri-implant diseases in comparison to healthy individuals. The secondary outcome measure was the ratio between pro-and anti-inflammatory modulators levels (IL-1/IL-10, IL-6/IL-10, IL-1/IL-1RA and RANKL/OPG) in sample tissue (mRNA) and biofluids (protein levels) of individuals with peri-implant diseases in comparison to healthy individuals.

### Literature search

Detailed search strategies were conducted on the PubMed, Cochrane Controlled Trials Registry, Web of Science, EMBASE and Scopus databases for publications up to March 2023. Grey literature was also searched through Google scholar. Search restrictions, including language and publication period, were not made. Publications were found using a combination of terms shown in supplementary materials. The publications found in all electronic databases was transferred to the EndNote Program™ X7 version (Thomson Reuters, New York, NY, USA) to remove duplicate references.

### Data selection and extraction

Two investigators (J.A.O. and R.O.A.) made the initial search for the evaluation of titles and abstracts independently, and the results were checked for agreement. The full text of the articles included based on title and abstract were independently read and evaluated based on the selection criteria (J.A.O. and R.O.A.). A discussion including a third investigator (S.C.P.) was reached for conflicting evaluations.

Two investigators (J.A.O. and R.O.A.) independently read all studies and extracted the following data: (i) the number of individuals comprised in each group; (ii) mean age and standard deviation of patients of each group; (iii) study groups (control, peri-implant mucositis and peri-implantitis); (iv) diagnostic criteria for peri-implant diseases; (v) assay method (RT-qPCR, ELISA, Multiplex); (vi) biological material evaluated (tissue sample or biofluids [PICF and saliva]); (vii) mediators evaluated in the study; and (viii) concentration of modulators molecules chosen to focus on this investigation, including the units for quantifying it. Relevant information from the selected studies according to the eligibility criteria is summarized in Table 1.

**Table 1:** Characteristics of studies and participants included in the systematic review according to the PECO´s principles

Author Year	Study Design	Sample Size	Mean age		Diagnostic criteria			Evaluation Method	Biological Sample	Mediators
			Control	Disease	Control	Disease #1	Disease #2			
Arıkan, Buduneli [34]	Cohort	CG: 79 DG1: 4 DG2: 3	53.5	DG1: 52.8 DG2: 66.3	No PD deeper than 4 mm, no suppuration, no plaque or gingival inflammation, and indicated no sign of bone loss in the radiographs	**Mucositis:** Implants with BOP, no suppuration or radiographic evidence of bone loss, and no PD deeper than 5 mm	**Peri-Implantitis:** Implants with PD deeper than 5 mm, BOP, suppuration, and radiographic evidence of crestal bone loss in at least one site	ELISA	PICF	RANKL, OPG
Arikan, Buduneli [35]	Case–control	CG: 21 DG: 18	52	56	Absence of PD deeper than 4 mm, an absence of suppuration, absence of plaque, absence of gingival inflammation in terms of BOP, and a lack of radiographic signs of bone loss	**Peri-implantitis:** Implants with the PD of at least one measurement site and frequently more than one—was = 5 mm with BOP and/or suppuration. Radiographic evidence of crestal bone loss involving at least three threads in at least one site but no more than half of the implant length	**-**	ELISA	PICF	RANKL, OPG
Ata-Ali, Flichy-Fernandez [8]	Cross-sectional	CG: 54 DG: 24	63,6	52	PD < 4 mm, absence of clinical signs of inflammation of the peri-implant mucosa, and without radiographic bone loss	**Peri-implantitis:** Implant with a PD = 4 mm and signs of acute peri-implantitis (loss of supporting bone as estimated on radiographs, BOP, or suppuration) and no implant mobility	**-**	Multiplex	PICF	IL-1β, IL-6, IL-10
Casado, Canullo [27]	Case–control	CG: 10 DG1: 10 DG2: 10	49.5	DG1: 52.8 DG2: 57.4	No clinical signs of inflammation in the peri-implant mucosa and no sign of bone loss in all regions	**Mucositis:** Implants with BOP, red mucosa and swelling spontaneous bleeding, but no radiographic signs of pathologic bone loss	**Peri-Implantitis:** Clinical signs of inflammation, including implant mobility and suppuration in some cases, and radiographic signs of bone loss	ELISA	PICF	IL-1β, IL-10
Chaparro, Sanz [36]	Cross-sectional	CG: 17 DG1: 19 DG2: 18	NI	DG1: NI DG2:NI	Absence of swelling, bleeding on probing, inflammation, and suppuration; besides the absence of increased probing depth and the absence of radiographic bone loss	**Mucositis:** Inflammation of the peri-implant soft tissues, without bone loss, but with bleeding on probing, swelling, and suppuration in some cases	**Peri-Implantitis:** Presence of inflammation, as in peri-mucositis, but with the presence of progressive bone loss	Multiplex	PICF	RANKL, OPG
Chaparro, Beltran [37]	Cross-sectional	CG: 7 DG1: 2 DG2: 10	73.8	DG1: 61.8 DG2: 67.8	Absence of visual signs of inflammation and bleeding on probing	**Mucositis:** Bleeding on probing and visual signs of inflammation	**Peri-Implantitis:** Presence of inflammation in the peri-implant mucosa and subsequent progressive loss of supporting bone	Multiplex	PICF	RANKL, OPG
Duarte, de Mendonça [38]	Case–control	CG: 10 DG1: 10 DG2: 15	49.1	DG1: 55.8 DG2: 55.8	No marginal bleeding, bleeding on probing, suppuration, and radiographic bone loss	**Mucositis:** Implants with marginal bleeding and/or bleeding on probing, and absence of radiographic bone loss and suppuration	**Peri-Implantitis:** Implants with PD deeper or equal to 5 mm, BOP and/or suppuration, radiographic bone loss involving at least three threads of the implant but no more than half of the implant length	ELISA	PICF	RANKL, OPG
Duarte, De Mendonça [28]	Longitudinal	CG: 11 DG1: 15 DG2: 10 12**	49.1	DG1: 55.8 DG2: 56.7 59.4**	Implants with PD ≤ 4 mm, without marginal bleeding, BOP, suppuration, and radiographic evidence of bone loss	**Mucositis:** Implants with marginal bleeding and/or BOP, absence of radiographic bone loss, and suppuration	**Peri-Implantitis:** Implants with PD ≥ 5 mm, with BOP and/or suppuration and radiographic bone loss involving four threads. **Severe Peri-Implantitis:** Implants with PD ≥ 5 mm with BOP and/or suppuration and radiographic bone loss involving more than four threads	RT-qPCR	Tissue	RANKL, OPG
Fonseca, Moraes Junior [39]	Case–control	DG1: 12 DG2: 10	NI	DG1:65 DG2:59,4	NI	**Mucositis:** Patients who showed inflamed sites with bone loss around the implants no deeper than the first implant’s thread and PD ≤ 3 mm	**Peri-Implantitis:** Patients who showed inflamed sites with at least one implant with bone loss around two or more threads of the implant and pocket depth ≥ 4 mm	Multiplex	PICF	IL-1β, IL-6, IL-10
Ghighi, Llorens [40]	Case–control	CG: 10 DG: 11	NI	NI	Patients underwent surgery for erupted third molar extraction and should not have either history of periodontitis or peri-implantitis according to clinical criteria of gingival bleeding, PD, and radiographic evidence of bone resorptions	**Peri-Implantitis:** Patients should present at least one dental implant in function with a titanium abutment, a PD ≥ 5 mm with BOP, and radiographic evidence of bone loss	-	Multiplex	Tissue	RANKL, OPG
Guncu, Akman [41]	Case–control	CG: 20 DG:27	NI	NI	Implants with Gingival Index = 0	**Mucositis:** Implantts with Gingival Index > 0	**-**	ELISA	PICF	IL-1β, IL-10, RANKL, OPG
Kandaswamy, Sakulpaptong [21]	Cross-sectional	CG: 25 implants DG1: 33 implants DG2: 59 implants	NI	DG1: NI DG2:NI	Absence of visual signs of inflammation and bleeding on probing	**Mucositis:** Bleeding on probing and visual signs of inflammation	**Peri-Implantitis:** Presence of inflammation in the peri-implant mucosa and subsequent progressive loss of supporting bone	Multiplex	PICF	IL-1β, IL-6, IL-10
Milinkovic, Djinic Krasavcevic [42]	Cross-sectional	CG:35 DG1:50 DG2:45	41,57	DG1: 55,22 DG2: 45,98	Absence of clinical signs of inflammation; absence of bleeding and/or suppuration on gentle probing; no increase in PD; absence of bone loss beyond crestal bone level changes resulting from initial bone remodeling	**Mucositis:** Bleeding and/or suppuration on gentle probing with or without increased PD; absence of continuing bone loss as observed on a radiograph: absenceof loss beyond crestal bone level changes resulting from initial bone remodeling	**Peri-Implantitis:** Presence of bleeding and/or suppuration on gentle probing; PD of ≥ 6 mm; bone levels ≥ 3 mm apical of the most coronal portion of the intraosseous part of the implant	RT-qPCR	PICF	IL-1β, IL-6, RANKL, OPG
Rakic, Lekovic [43]	Cross-sectional	CG: 25 DG: 23	36	DG: 48	When there was no implant with signs of inflammation (no BOP), no presence of pockets (PD ≤ 3 mm), and without radiographically evidenced bone loss	**Peri-Implantitis:** Presence of PPD ≥ 5 mm, with positive BOP and recorded RXBL ≥ 2 threads compared to the radiograph taken at the time of prosthetic replacement	**-**	ELISA	PICF	RAKL, OPG
Rakic, Struillou [44]	Cross-sectional	CG: 58 DG1: 52 DG2: 54	54,66	DG1: 57,39 DG2: 51,83	These controls were defined by PD ≤ 3 mm, no BOP, and no BL evidenced by radiograph	**Mucositis:** Cases were defined by the presence of peri-implant PD ≥ 3 mm, with positive BOP and absence of radiographic BL compared with the radiograph taken at the time of prosthetic replacement	**Peri-Implantitis:** Cases were defined by the presence of PD ≥ 5 mm, with positive BOP and recorded radiographic BL involving at least two threads compared with the radiograph taken at the time of the prosthetic replacement	ELISA	PICF	RANKL, OPG
Rakic, Petkovic-Curcin [45]	Case–control	CG: 189DG: 180	49.4	53.2	When there was no implant with signs of inflammation (no BOP), no presence of pockets (PD ≤ 3 mm), and without radiographically evidenced bone loss	**Peri-Implantitis:** Presence of PD ≥ 5 mm, with positive bleeding on probing and recorded radiographic bone loss involving ≥ 2 threads compared to the radiograph taken at the time of prosthetic replacement	**-**	ELISA	PICF	RANKL. OPG
Rakic, Monje [46]	Case–control	CG: 126 DG1: 57 DG2: 69	NI	DG1: 52.5 DG2: 53.14	Implants with negative BOP or BOP positive in 1/6 sites being considered the consequence of trauma, with PD < 3 mm and without evidence of radiological bone loss	**Mucositis:** Implants with negative BOP or BOP positive in 1/6 sites being considered the consequence of trauma, with PD < 3 mm and without evidence of RXBL	**Peri-Implantitis:** PD ≥ 5 mm, BOP > 1, and RXBL involving ≥ 2 mm compared to the radiograph taken at the time of prosthetic loading	ELISA	PICF	RANKL, OPG
Song, Jiang [47]	Cross-sectional	CG: 14 implants DG: 14 implants	NI	NI	With an absence of soft tissue inflammation and further additional bone loss following initial healing according to radiographic examination at baseline and at follow-up	**Peri-Implantitis:** With bone loss and increasing PD following initial healing, or with MBL ≥ 3 mm and PD ≥ 6 mm without previous examination data	-	Multiplex	PICF	IL1β x IL1-Ra
Severino, Napimoga [48]	Case–control	CG: 20 implants DG: 20 implants	52.27	47.5	Implants with PD of 0–3 mm, without marginal bleeding, suppuration, or bone loss	**Mucositis:** Implants with PD of 0–3 mm, with marginal bleeding, without suppuration or bone loss	**-**	ELISA	PICF	IL-6, IL-10
Severino, Beghini [4]	Case–control	CG: 10 DG1: 20 DG2: 20	75.20	DG1: 60.88 DG2: 60.81	Implants with PD of 0–3 mm, without marginal bleeding, suppuration, or bone loss	**Mucositis:** Implants with PD of 0–3 mm, with marginal bleeding, without suppuration or bone loss	**Peri-Implantitis:** Implants with marginal bleeding, with PD greater than 3 mm, and bone loss in at least one site of the implant	ELISA	PICF	IL-6, IL-10
Teixeira, Lira‐Junior [33]	Case–control	CG: 9 DG1: 10 DG2: 14	66.7	DG1: 59.8 DG2: 59.9	-	**Mucositis:** Clinically inflamed sites and no significant radiographic bone loss	**Peri-Implantitis:** Inflamed sites and bone loss involving two or more implant threads	Multiplex	PICF	IL-1β, IL-6, IL-10
Yakar, Guncu [49]	Case–control	CG: 25 DG: 27	50.64	55.85	No sign of inflammation, no sites with less than 4 mm probing depth, and no evident radiographic bone loss at a relevant implant	**Peri-Implantitis:** Presence of at least one peri-implant site with PD of ≥ 6 mm accompanied by at least one of the other signs as radiographic bone loss purulent exudate or bleeding	**-**	ELISA	PICF	RANKL, OPG

*CG* Control Group, *DG* Disease Group, *IL-* Interleukin, *RANKL* Receptor Activator of Nuclear Factor Kappa-B Ligand, *OPG* Osteoprotegerin, *BOP* Bleeding on Probing, *PD* Probing Depth, *CAL* Clinical Attachment Level, *NI* Not informed, *RXBL* Radiological bone loss, *PICF* Peri-Implant Crevicular Fluid, *MBL* Marginal bone loss, ** Data for severe peri-implantitis.

### Quality assessment

Two authors (J.A.O. and R.O.A.) separately evaluated the quality of the included studies. No disagreement between both evaluators were observed. The Newcastle–Ottawa scale was used to evaluated case–control studies [50]. Using this scale, the studies were judged on three general perspectives: the selection of the study groups [case definition (peri-implantitis or peri-implant mucositis) with independent validation; representativeness of the cases: consecutive or obviously representative series of cases; selection of controls: community controls; definition of controls: no history of disease], the comparability of the groups [study controls for smoke; study controls for systemic disease], and the ascertainment of either the exposure or outcome of interest for case–control [ascertainment of exposure: secure record; same method of ascertainment for cases and controls; nonresponse rate: same rate for both groups]. Studies with the highest quality received nine points. A total score lower than 3 was classified as “low quality”, a score of 4 or 5 was classified as “moderate quality,” and a score of 6 or more was classified to be “high quality”.

For cross-sectional studies, the Risk of Bias Assessment Tool for Nonrandomized Studies scale (RoBANS) was used [51]. The RoBANS comprises 6 domains including the selection of participants (selection bias caused by inadequate participants selection), confounding variables [selection bias caused by inadequate confirmation and consideration of confounding variable (smoking habits and systemic diseases)], measurement of exposure (performance bias caused by inadequate measurement of exposure), blinding of outcome assessment (Detection bias caused by inadequate blinding of outcome assessment), incomplete outcome data (Attrition bias caused by inadequate handling of incomplete outcome data) and selective outcome reporting (Reporting bias caused by selective outcome reporting). The domains were classified with low, unclear or high risk of bias.

### Data synthesis- meta-analysis

Only studies using the same assay method was included in the meta-analysis. Consequently, for meta-analysis evaluating the mediator´s levels in PICF (protein levels), only studies using ELISA were included. The measure unit used was pg/ml. Two studies [35, 41] used pg/μL and one study [41] used pmol/μL as measure unit. The mediators’ levels from these studies were converted to pg/ml using an online conversion website (http://www.endmemo.com/convert/). The effect size was estimated and reported as the mean difference. The 95% confidence interval was estimated for each mediator, and the pooled effect was determined significant if two-sided *p*-values < 0.05 were obtained. The forest plots were produced using statistical software (Review Manager [RevMan], Version 5.1. Copenhagen: The Nordic Cochrane Centre, The Cochrane Collaboration, 2011).

Forest plots for each meta-analysis showed the raw data (i.e., means, standard deviations, and sample sizes), point estimates (displayed as blocks) and confidence intervals (displayed as lines) for the chosen effect. Moreover, the heterogeneity statistics, the total number of participants per group, the overall average effect (mean difference and Z-statistics), and percent weight assigned to each study were also showed [52]. Chi-square (*x*^*2*^) and inconsistency index (*I*^*2*^) tests were used to evaluate the heterogeneity of the studies included in this meta-analysis. The *I*^*2*^ value was shown as a percentage of the total variation across studies. When *I*^*2*^ > 50%, the assumption of homogeneity was deemed invalid, and the random-effects model (DerSimonian-Laird method) was applied; otherwise, the fixed model (Mantel–Haenszel method) was used for the meta-analysis [53]. Publication bias was evaluated by using funnel plots.

## Results

**Fig. 1:**
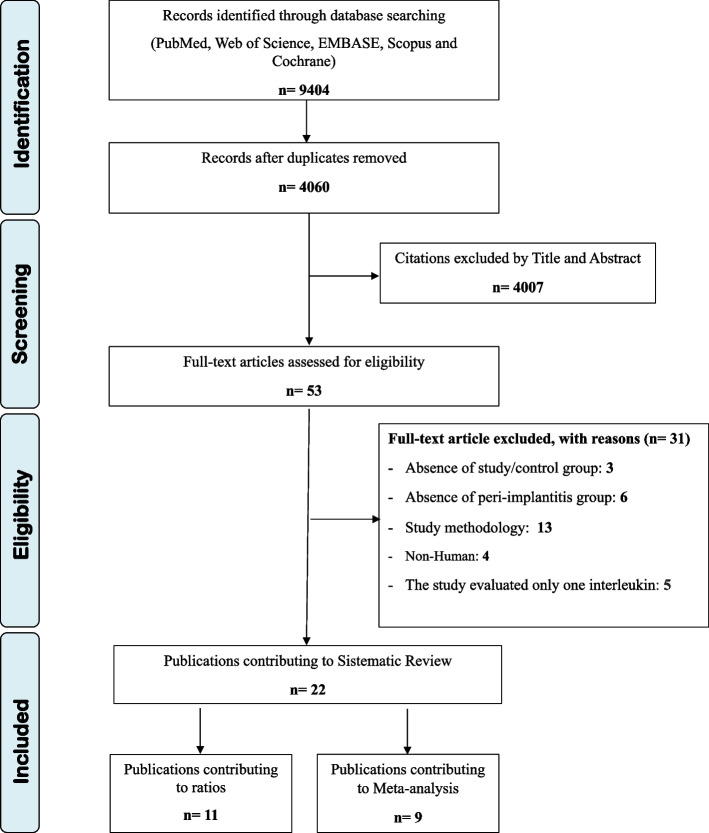
Flow chart of the search strategy of the study

In electronic search a total of 9404 hits were found, being 4060 unique citations. A total of 53 publications were evaluated as full-text copies and 31 of these publications were excluded based on priori criteria (Fig. 1). The exclusion motivation for each excluded study was shown in Table S1 (supplementary materials). The remaining 22 publications were included in the systematic review (qualitative analysis). From those 22 publications, 11 studies included the ratios between RANKL/OPG and OPG/RANKL (qualitative analysis) and 9 publications composed the meta-analyses (quantitative analysis).

### Qualitative analysis

For quality assessment analysis, all case–control studies (12 studies) were considered as high quality (Table 2). Concerning cross-sectional studies (10 studies), all studies was classified with low risk of bias for domains 1, 3 and 6. For domain 2 (confounding variable presence), seven studies were classified as high risk of bias and three studies as low risk of bias. For domain 4, only one study described information about outcome assessment blinding (Table 3).

**Table 2:** Quality assessment of the case–control studies using the Newcastle Ottawa scale

Autor	Year	Criterion Scores			Total score
		Selection	Comparability	Exposure	
Arikan	2011	1–2-3–4	1–2	1–2-3	9—high quality
Casado	2013	1–4	1–2	1–2-3	7—high quality
Duarte	2009	1–2-4	1–2	1–2-3	8—high quality
Fonseca	2012	1–2-3–4	1–2	1–2-3	9—high quality
Ghigh	2017	1–2-4	1–2	1–2-3	8—high quality
Guncu	2012	1–3-4	1–2	1–2-3	8—high quality
Rakic	2015	1–2-3–4	1–2	1–2-3	9—high quality
Rakic	2020	1–2-3–4	1–2	1–2-3	9—high quality
Severino	2011	1–2-3–4	1–2	1–2-3	9—high quality
Severino	2016	1–2-3–4	1–2	1–2-3	9—high quality
Teixeira	2016	1–4	1–2	1–2-3	7—high quality
Yakar	2018	1–2-3–4	1–2	1–2-3	9—high quality

Selection: 1. Is the Case Definition Adequate? Yes, with independent validation; 2. Representativeness of the Cases: Consecutive or Obviously Representative Series of Cases; 3. Selection of Controls: Community Controls; 4. Definition of Controls: No History of Disease (endpoint); Comparability: 1. Study Controls for smoke; 2. Study Controls for systemic disease; Exposure: 1. Ascertainment of Exposure: Secure Record; 2. The same method of: Yes; 3. Nonresponse rate: same rate for both groups

**Table 3:** Quality assessment of the cross-sectional studies using the non-randomized studies scale

Author name	Year	Domain					
		**1**	**2**	**3**	**4**	**5**	**6**
Arikan	2008	High	Low	High	Unclear	High	High
Ata-Ali	2015	High	Low	High	Unclear	High	High
Chaparro	2020	High	Low	High	Unclear	High	High
Chaparro	2022	High	Low	High	Unclear	High	High
Duarte	2009	High	High	High	Unclear	High	High
Kandaswamy	2022	High	Low	High	High	High	High
Milinkovic	2021	High	Low	High	Unclear	High	High
Rakic	2013	High	High	High	Unclear	High	High
Rakic	2014	High	High	High	Unclear	High	High
Song	2022	High	Low	High	Unclear	High	High

Domain 1: Selection bias caused by inadequate selection of participants; Domain 2: Selection bias caused by inadequate confirmation and consideration of confounding variables (smoke habits and systemic diseases); Domain 3: Performance bias caused by inadequate measurement of intervention (exposure); Domain 4: Detection bias caused by inadequate blinding of outcome assessment; Domain 5: Attrition bias caused by inadequate handling of incomplete outcome data; Domain 6: Reporting bias caused by selective outcome reporting

Tables S2-S19 (supplementary materials) support the systematic review which utilized qualitative analysis. Data from each study is summarized presented, only intending to show the levels of pro-and anti-inflammatory cytokines (IL-1 and IL-10; IL-6 and IL-10) and osteoclastogenesis-related factors (RANKL and OPG) in a tissue sample (gene expression) and biofluids (protein measurement). No studies evaluating the mediators in blood were found. Because the methodologies to assess protein measurement are different, these tables did not intend to compare the methods, but just to widely present the reported levels of the peri-implantitis modulators. Table S20 (supplementary materials) reports the data extracted about the limitations and funding data of included studies.

### Findings of the IL-1 and IL-10 levels

For IL-1β and IL-10 levels, all studies included in qualitative analysis evaluated both cytokines only in PICF (Table 4; Tables S2-S4). Higher levels of both cytokines were found in individuals with mucositis [21, 41] and peri-implantitis in comparison to healthy individuals [8, 21, 40] (Table 4; Tables S2 and S3). One study showed higher IL-1β levels and lower levels of IL-10 in individuals with mucositis and peri-implantitis in comparison to healthy [27] (Table 4; Tables S2 and S3). Comparing mucositis and peri-implantitis, three studies showed higher IL-1β levels and lower levels of IL-10 in peri-implantitis individuals [21, 27, 39] (Table 4; Table S4). One study showed lower levels of both cytokines in peri-implantitis individuals [33] (Table 4; Table S4).

**Table 4:** Summarized findings of qualitative analysis (systematic review) for IL-1 versus IL-10, IL-6 versus IL-10, IL-1 versus IL-1Ra, and RANKL versus OPG

*IL-1 versus IL-10*			
**Cytokine**	**Sample type**	**Peri-implant mucositis/peri-implantitis versus control**	**Studies**
IL-1β	PICF	Higher in disease	Guncu et al. 2012 [41], Casado et al. 2013 [27], Kandaswamy et al. 2022 [21]
IL-10	PICF	Higher in disease	Guncu, Akman et al. 2012 [41], Kandaswamy et al. 2022 [21]
IL-10	PICF	Lower in disease	Casado et al. 2013 [27]
**Cytokine**	**Sample type**	**Peri-implant mucositis versus peri-implantitis**	**Studies**
IL-1β	PICF	Higher in peri-implantitis	Casado et al. 2013 [27], Fonseca et al. 2014 [39], Kandaswamy et al. 2022 [21]
IL-1β	PICF	Lower in peri-implantitis	Teixeira et al. 2017 [33]
IL-10	PICF	Lower in peri-implantitis	Casado et al. 2013 [27], Fonseca, Moraes et al. 2014 [39], Teixeira et al. 2017 [33], Kandaswamy et al. 2022 [21]
***IL-1 versus IL-1Ra***			
**Cytokine**	**Sample type**	**Peri-implantitis versus control**	**Studies**
IL-1β	PICF	Higher in disease	Song et al. 2022 [47]
IL-Ra	PICF	Lower in disease	Song et al. 2022 [47]
***IL-6 versus IL-10***			
**Cytokine**	**Sample type**	**Peri-implant mucositis/peri-implantitis versus control**	**Studies**
IL-6	PICF	Higher in disease	Severino et al. 2011 [48], Fonseca et al. 2014 [39], Ata-Ali et al. 2015 [8], Severino et al. 2016 [4], Kandaswamy et al. 2022 [21]
IL-6	Saliva	Higher in disease	Severino et al. 2016 [48]
IL-10	PICF	Higher in disease	Ata-Ali et al. 2015 [8], Severino et al. 2016 [4], Kandaswamy et al. 2022 [21]
IL-10	PICF	Lower in disease	Severino et al. 2011 [48], Fonseca et al. 2014 [39]
IL-10	Saliva	Lower in disease	Severino et al. 2016 [4]
**Cytokine**	**Sample type**	**Peri-implant mucositis versus peri-implantitis**	**Studies**
IL-10	PICF	Lower in peri-implantitis	Severino et al. 2016 [4], Teixeira et al. 2017 [33], Kandaswamy et al. 2022 [21]
***RANKL versus OPG***			
**Cytokine**	**Sample type**	**Peri-implant mucositis/peri-implantitis versus control**	**Studies**
RANKL	PICF	Higher in disease	Guncu et al. 2012 [41], Rakic et al. 2013 [43], Rakic et al. 2014 [44], Rakic et al. 2015 [45], Yakar et al. 2019 [49], Chaparro et al. 2020 [36], Rakic et al. 2020 [46], Milinkovic et al. 2021 [42], Chaparro et al. 2022 [37]
RANKL	Tissue sample	Higher in disease	Duarte, De Mendonça et al. 2009 [28] Ghighi, Llorens et al. 2018 [53]
OPG	PICF	Higher in disease	Guncu et al. 2012 [41], Rakic et al. 2013 [43], Rakic et al. 2014 [44], Rakic et al. 2015 [45], Yakar et al. 2019 [49], Chaparro et al. 2020 [36], Rakic et al. 2020 [46], Milinkovic et al. 2021 [42], Chaparro et al. 2022 [37]
OPG	Tissue sample	Lower in disease	Duarte et al. 2009 [28], Ghighi et al. 2018 [53]
**Cytokine**	**Sample type**	**Peri-implant mucositis versus peri-implantitis**	**Studies**
RANKL	PICF	Higher in peri-implantitis	Arıkan et al. 2008 [34], Duarte et al. 2009 [38] Rakic et al. 2014 [44], Milinkovic et al. 2021 [42]
RANKL	PICF	Lower in peri-implantitis	Rakic, Monje et al. 2020 [46]
RANKL	Tissue sample	Higher in peri-implantitis	Duarte et al. 2009 [38]
OPG	PICF	Higher in peri-implantitis	Arıkan et al. 2008 [34], Rakic et al. 2014 [44]
OPG	PICF	Lower in peri-implantitis	Duarte et al. 2009 [38], Chaparro et al. 2020 [36], Rakic et al. 2020 [46], Milinkovic et al. 2021 [42], Chaparro et al. 2022 [37]
OPG	Tissue sample	Higher in peri-implantitis	Duarte et al. 2009 [28]

*PICF* peri-implant crevicular fluid

### Findings of the IL-1 and IL-Ra levels

For IL-1β and IL-Ra levels, only one study [47] was included and observed higher levels of IL-1β and lower levels of IL-Ra in PICF of individuals with peri-implantitis in comparison to healthy individuals (Table 4; Table S5).

### Findings of the IL-6 and IL-10 levels

Higher IL-6 and IL-10 levels in PICF of individuals with mucositis in comparison to healthy individuals were observed [4, 21] (Table 4; Table S6). Three studies also showed higher IL-6 and IL-10 levels in PICF of individuals with peri-implantitis in comparison with healthy individuals [4, 8, 21] (Table 4; Table S7). Contrariwise, two studies observed higher IL-6 levels and lower levels of IL-10 in peri-implantitis in comparison to healthy individuals [39, 48] (Table 4; Table S7). Comparing mucositis and peri-implantitis, three studies were included and showed lower levels of IL-10 in peri-implantitis subjects [4, 21, 33] (Table 4; Table S8).

Considering the evaluation in the saliva, higher levels of IL-6 and IL-10 were found in individuals with mucositis in comparison to healthy individuals [4] (Table 4; Table S9). Higher levels of IL-6 and lower levels of IL-10 were found in peri-implantitis individuals in comparison to mucositis and healthy individuals [4] (Table 4; Table S10 and S11).

### Findings of the RANKL and OPG levels

In general, the studies showed higher levels of RANKL and OPG in PICF of individuals with mucositis [37, 41, 42, 46] and peri-implantitis [36, 43–46, 49] in comparison to healthy individuals (Table 4; Tables S12 e S13). Seven studies compared RANKL and OPG levels in PICF of individuals with mucositis and peri-implantitis [34, 36–38, 42, 44, 46] (Table 4; Table S14); from them, six studies [34, 38, 42, 44] found higher levels of RANKL in peri-implantitis individuals. For OPG, higher levels in peri-implantitis individuals were observed in two studies [34, 44] and lower levels in peri-implantitis individuals were found in five studies [36–38, 42, 46].

For tissue samples obtained from peri-implant pocket sites, higher levels of RANKL were found in individuals with peri-implant mucositis [28] and peri-implantitis compared with healthy individuals (Table 4; Tables S15 and S16). For OPG, lower levels were found in individuals with mucositis [28] and peri-implantitis [28, 40] in comparison to healthy individuals (Table 4; Tables S15 and S16). Higher levels of RANKL and OPG were found in individuals with peri-implantitis in comparison to mucositis individuals [28] (Table 4; Table S18). Only one study [28] divided the tissue samples in healthy, mucositis, initial peri-implantitis (involving four threads) and severe peri-implantitis (involving more than four threads). Severe peri-implantitis individuals showed higher levels of RANKL and OPG in comparison to health and mucositis individuals (Tables S17 and S19).

#### Findings of ratios between osteoclastogenesis-related factors

Higher RANKL/OPG ratios were observed in PICF from individuals with mucositis [36, 44, 46] and peri-implantitis [35, 36, 40, 43–46, 49] in comparison to healthy individuals (Table 5). Also, higher RANKL/OPG ratio levels were showed in PICF from individuals with peri-implantitis in comparison to mucositis individuals [44, 46] (Table 5).

**Table 5:** RANKL: OPG and OPG: RANKL ratio in peri-implant crevicular fluid and tissue samples from mucositis, peri-implantitis, and health patients

RANKL: OPG			
**Control versus Mucositis** Peri-implant crevicular fluid			
Author, Year	Evaluation Method	Control	Disease
Chaparro, Sanz et al. (2020) [36]	MULTIPLEX	0.29 (0.26 – 0.43)	0.37 (0.24 – 0.58)
Guncu, Akman et al. (2012) [41]	ELISA	2,65 ± 1,64	1,71 ± 0,89
Rakic, Struillou et al. (2014) [44]	ELISA	0,72 ± 0,63	0,92 ± 1,32
Rakic, Monje et al. (2020) [46]	ELISA		HIDS***
**Control versus Peri-implantitis** Peri-implant crevicular fluid			
Author, Year	Evaluation Method	Control	Disease
Arikan, Buduneli et al. (2011) [35]	ELISA	0,4 ± 0,2	0,8 ± 0,9
Chaparro, Sanz et al. (2020) [36]	MULTIPLEX	0.29 (0.26 – 0.43)	0.31 (0.21 – 0.56)
Ghighi, Llorens et al. (2018) [40]	MULTIPLEX		HIDS**
Rakic, Lekovic et al. (2013) [43]	ELISA	0,81 ± 0,61	1,01 ± 1,17
Rakic, Struillou et al. (2014) [44]	ELISA	0,72 ± 0,63	1,01 ± 1,23
Rakic, Petkovic-Curcin et al. (2015) [45]	ELISA	0,40 ± 0,33	1,51 ± 1,14
Rakic, Monje et al. (2020) [46]	ELISA		HIDS***
Yakar, Guncu et al. (2019) [49]	ELISA	0,0153 ± 0,0171	0,0234 ± 0,0244
**Mucositis versus Peri-implantitis** Peri-implant crevicular fluid			
Author, Year	Evaluation Method	Mucositis	Peri-implantitis
Chaparro, Sanz et al. (2020) [36]	MULTIPLEX	0.37 (0.24 – 0.58)	0.31 (0.21 – 0.56)
Rakic, Struillou et al. (2014) [44]	ELISA	0,92 ± 1,32	1,01 ± 1,23
Rakic, Monje et al. (2020) [46]	ELISA		HIPS^**^
**OPG: RANKL**			
**Control versus Mucositis** Peri-implant crevicular fluid			
Author Year	Evaluation Method	Control	Disease
Duarte, de Mendonça et al. (2009) [38]	ELISA	2,79 ± 2,08	1,56 ± 0,96
**Control versus Peri-implantitis** Peri-implant crevicular fluid			
Author Year	Evaluation Method	Control	Disease
Duarte, de Mendonça et al. (2009) [38]	ELISA	2,79 ± 2,08	1,04 ± 0,76
Tissue sample			
Author Year	Evaluation Method	Control	Disease
Duarte, De Mendonça et al. (2009) [28]	PCR	20 ± 11,6	1,2 ± 0,7
**Mucositis versus Peri-implantitis** Peri-implant crevicular fluid			
Author Year	Evaluation Method	Mucositis	Peri-implantitis
Duarte, de Mendonça et al. (2009) [38]	ELISA	1,56 ± 0,96	1,04 ± 0,76

*HIDS* Higher in Diseased Subjects, *HIPS* Higher in Peri-implantitis Subjects

^**^p < 0.01

^***^p˂0.00,1

In the different analyses of the OPG/RANKL ratio, lower levels were observed in PICF from individuals with mucositis and peri-implantitis in comparison to healthy individuals [38] and individuals with peri-implantitis in comparison to mucositis [38] (Table 5). For tissue samples, one study [28] found a lower OPG/RANKL ratio in peri-implantitis individuals in comparison to healthy individuals (Table 5).

### Meta-analysis

**Fig. 2:**
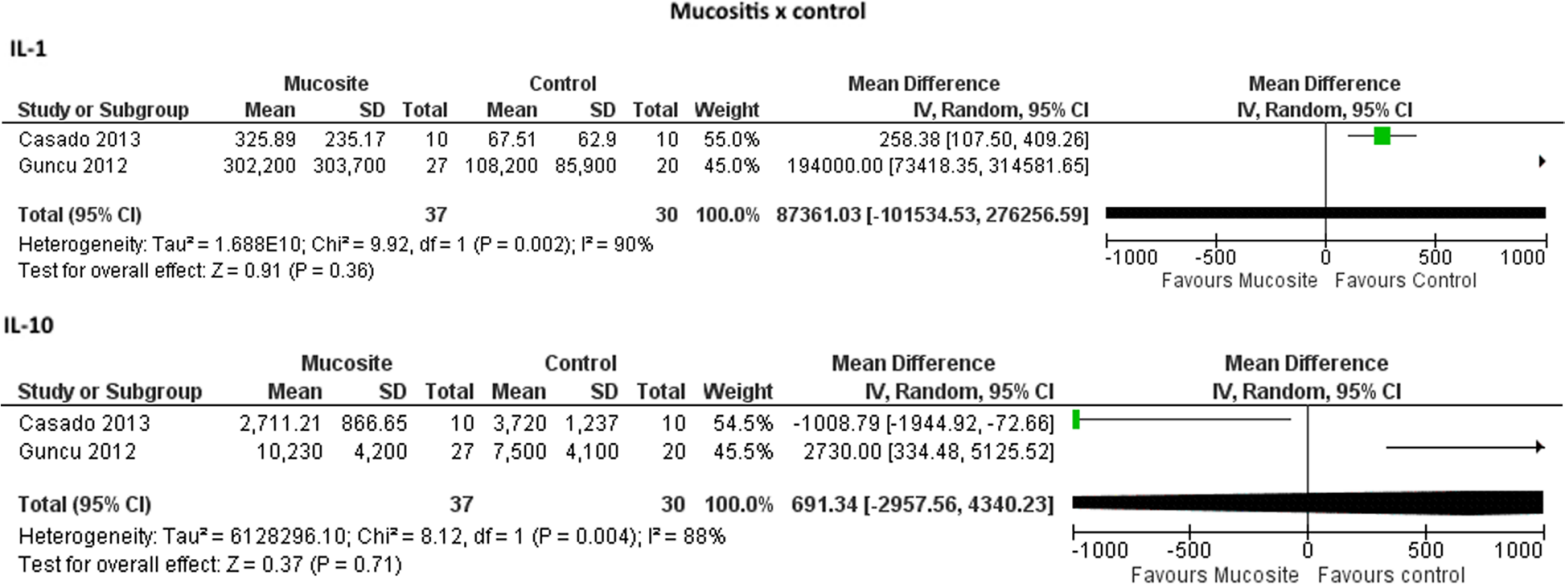
Meta-analyses forest plots of IL-1 and IL-10 levels in PICF found by ELISA (pg/mL) in individuals with mucositis in comparison with controls

**Fig. 3:**
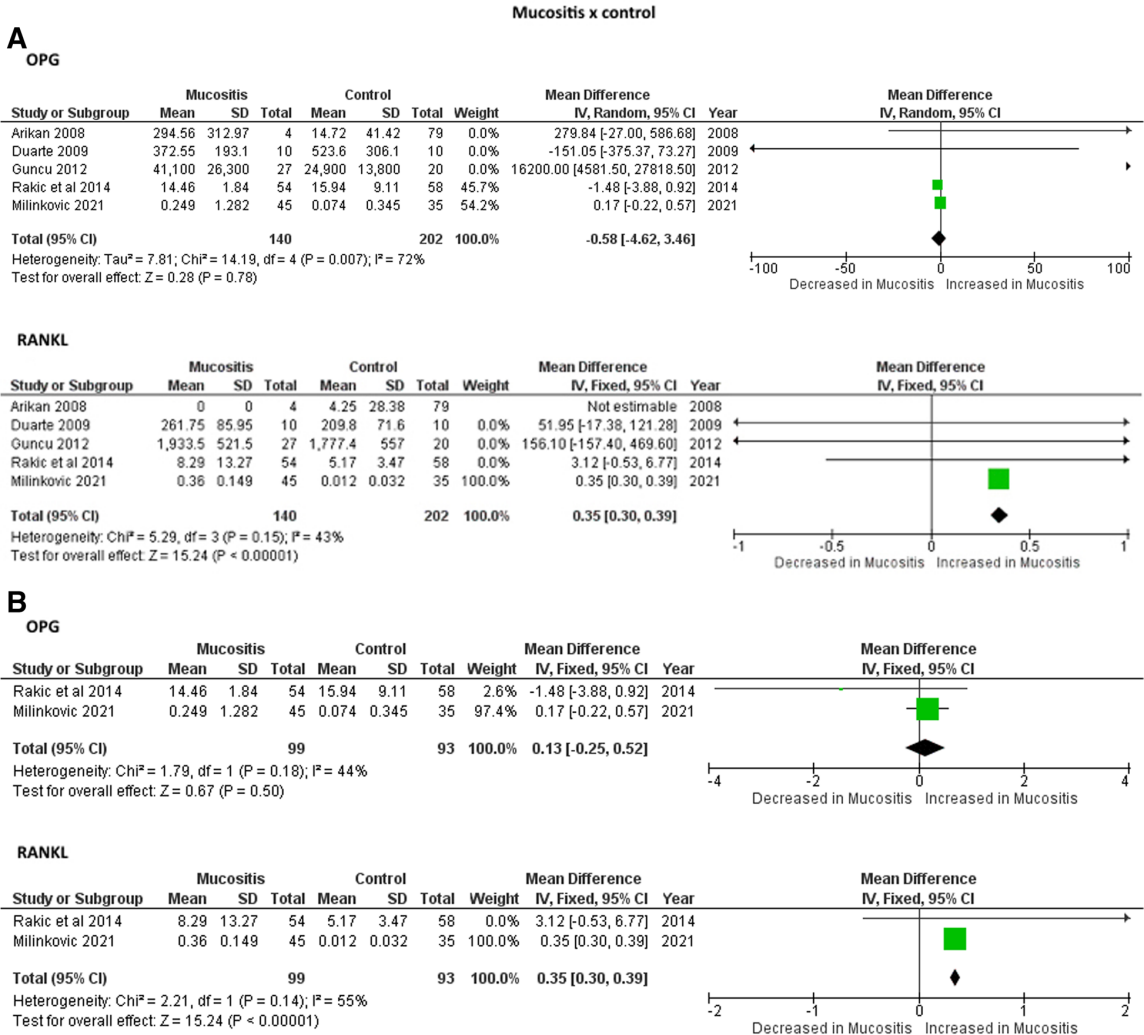
Meta-analyses forest plots of RANKL and OPG levels in PICF found by ELISA (pg/mL) in individuals with mucositis in comparison with controls. A: Including studies with measure unit conversion; B: Without studies with measure unit conversion

**Fig. 4:**
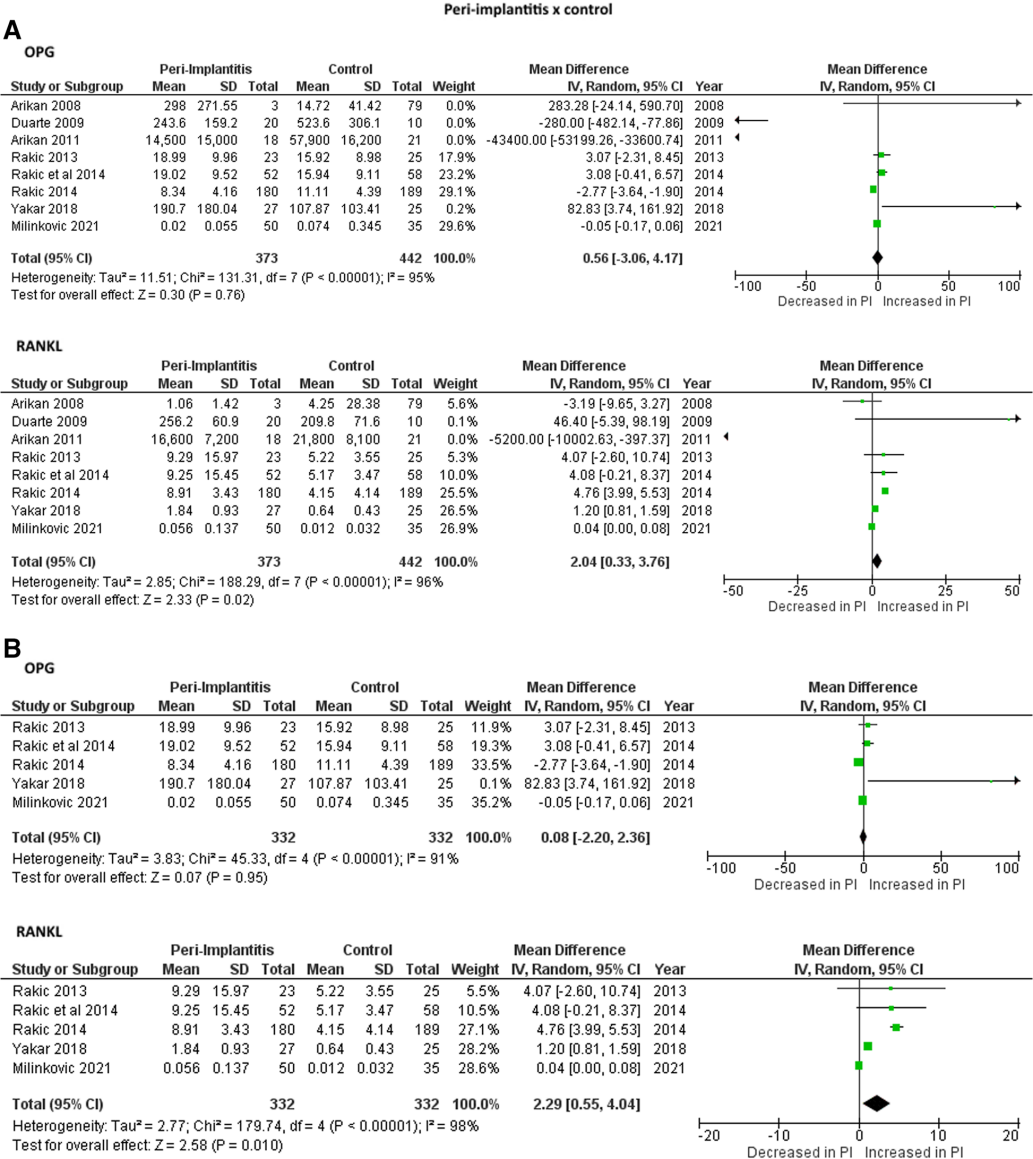
Meta-analyses forest plots of RANKL and OPG levels in PICF found by ELISA (pg/mL) in individuals with peri-implantitis in comparison with controls. A: Including studies with measure unit conversion; B: Without studies with measure unit conversion

**Fig. 5:**
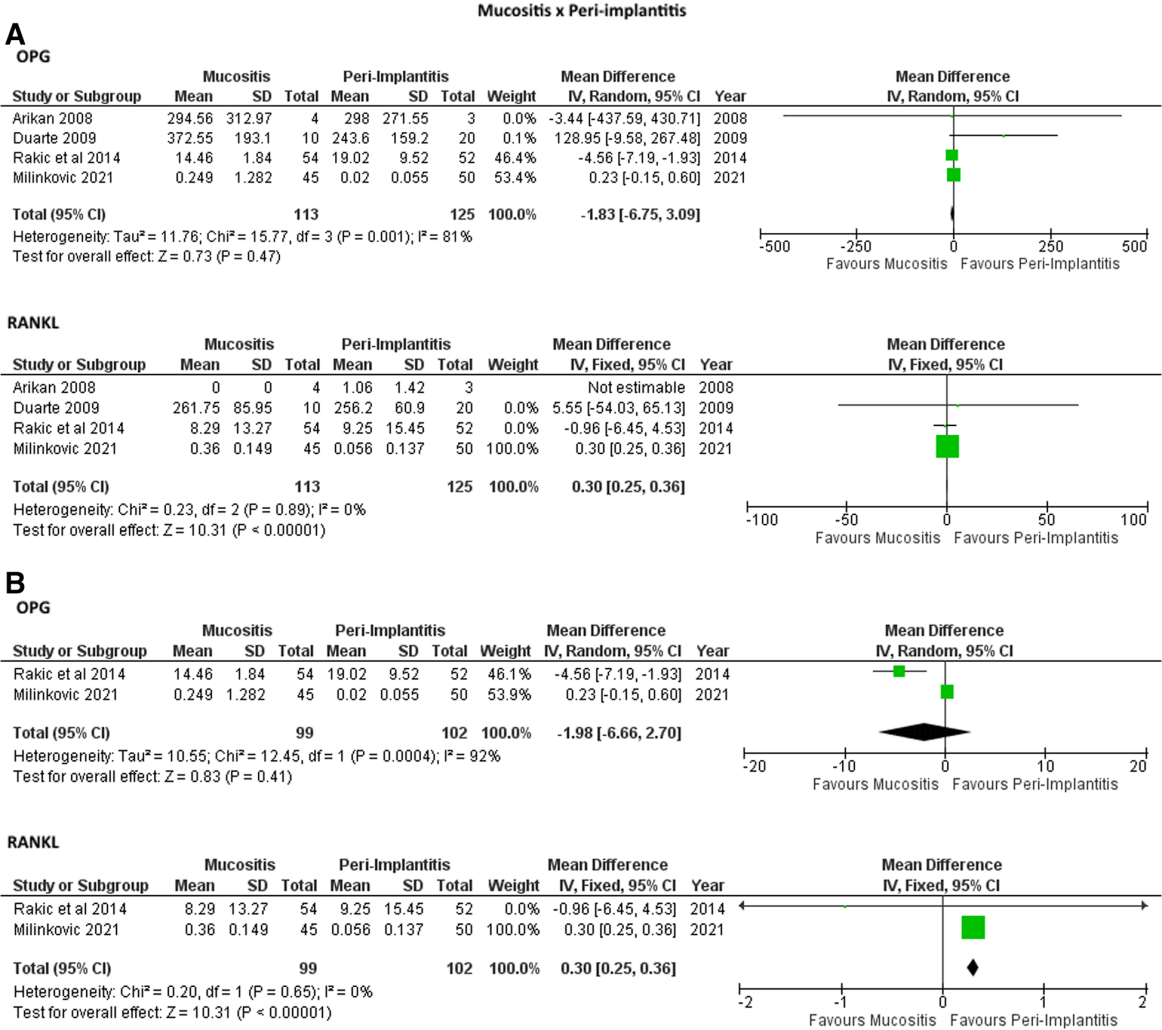
Meta-analyses forest plots of RANKL and OPG levels in PICF found by ELISA (pg/mL) in individuals with peri-implantitis in comparison with mucositis. A: Including studies with measure unit conversion; B: Without studies with measure unit conversion

Figure 2 show the meta-analysis results in which no significant differences were found in the IL-1 and IL-10 levels in PICF of mucositis individuals in comparison to healthy controls. Higher levels of RANKL were found in PICF of mucositis and peri-implantitis individuals in comparison to healthy controls in studies with (Fig. 3A and 4A) and without measure unit conversion (Fig. 3B and 4B). However, no differences were observed for OPG levels in PICF of mucositis and peri-implantitis individuals in comparison to healthy controls in studies with (Fig. 3A and 4A) and without measure unit conversion (Fig. 3B and 4B). For peri-implantitis individuals in comparison to mucositis, higher levels of RANKL were found in individuals with peri-implantitis considering only the studies without measure unit conversion (Fig. 5B). For the other analysis, no differences were observed for RANKL and OPG levels in PICF of peri-implantitis individuals in comparison to mucositis (Fig. 5).

## Discussion

Even though several studies investigated the peri-implant disease process, the association between pro and anti-inflammatory cytokines and osteoclastogenesis-related factors in healthy and diseased individuals seems to be still uncertain. Pro-inflammatory cytokines appear to stimulate a disproportionate inflammatory response that prejudices osseointegration success [27, 54]. The pro-inflammatory cytokines should be regulated by anti-inflammatory mediators, such as the IL-10, in an orchestrated and balanced way to adequately promote osseointegration [27]. Therefore, it seems reasonable to evaluate whether there would be disequilibrium between pro and anti-inflammatory cytokines, as well as between osteoclastogenesis-related factors, with the predominance of pro-inflammatory mediators, which could trigger a destructive reaction affecting the peri-implant disease progression and severity [27, 28]. Hence, we developed this systematic review with meta-analysis to better understand the complex networks of mediators involved in the inflammatory peri-implant disease pathogenesis.

In this meta-analysis, no differences were found in the IL-1β and IL-10 levels in PICF of individuals with mucositis in comparison to healthy individuals. Unlike, higher levels of both cytokines were found in individuals with peri-implantitis in comparison to healthy individuals [8, 40] in the qualitative analysis. This result is expected based on the role of IL-1 and IL-10 in the host's immune response. IL-1β production induces the release of a cascade of inflammatory mediators that result in soft and hard tissue destruction [27]. It has been shown that IL-1 plays an important role in the bone resorption associated with periodontitis inflammation by stimulating osteoclastogenesis [55, 56]. On the other hand, IL-10 acts suppressing macrophage activation and the production of the pro-inflammatory cytokines including TNF, IL-6 and IL-1 [55, 57–59]. In this way, IL-10 can act limiting the duration and magnitude of the immune and inflammatory responses [60–62].

In the same cascade way, the IL-6 production up-regulates the IL-1β and TNF-α production that may produce an inflammation amplification loop [63, 64] with a subsequent increase of RANKL expression [63], leading to increased bone resorption [48]. In the qualitative analysis, higher IL-6 levels in PICF and saliva of individuals with mucositis and peri-implantitis in comparison to health individuals were observed. Unlike, in general, the IL-10 levels in PICF and saliva were reduced in peri-implantitis disease in comparison to health and mucositis status. Collectively, these results suggest that the lower IL-10 levels in peri-implantitis individuals result in higher IL-6 cytokines levels potentially promoting a destructive inflammatory response around dental implants.

As revised by Cavalla, Letra [65], proinflammatory cytokines directly modulate RANKL and OPG expression and consequently drive inflammatory lesion progression, along with pro-osteoclastogenic support provided by T and B cells. It is known that the RANKL binds directly to RANK on the surface of preosteoclasts and osteoclasts, stimulating both the differentiation of osteoclast progenitors and the activity of mature osteoclasts [66, 67]. Conversely, OPG is a soluble molecule inhibiting osteoclast differentiation [34]. In both qualitative and quantitative analysis, higher RANKL levels were observed in PICF of peri-implantitis individuals in comparison to health and peri-implant mucositis in the present review. Therefore, based on the studies included in this review, it can be speculated that local upregulation of IL-1β, IL-6 and RANKL levels are linked with the local signs of inflammation in peri-implant tissues since they increase the osteoclast differentiation pathway. In addition, a higher RANKL/OPG ratio (as well as a lower OPG/RANKL ratio) was also observed in the PICF of peri-implantitis individuals in comparison to health and peri-implant mucositis. The results observed by the analyses of ratio levels suggested upregulation of RANKL and down-regulation of OPG, favoring the peri-implant bone resorption [28]. Also, up-regulated RANKL/OPG ratio was previously described in osteoblastic cells and periodontal ligament cells in response to immune cell-derived inflammatory cytokines and bacterial components [32].

Histopathology differences between periodontitis and peri-implantitis lesions are well accepted. Peri-implantitis inflammatory lesions are characteristically larger, with a higher density of plasma cells, neutrophils, and macrophages [68]. As a consequence, peri-implantitis is commonly identified to be more destructive than periodontitis [69] with more rapid progression and less predictable treatment outcomes [68]. A superior quantity of bone resorption has been observed around implants in comparison to natural teeth in experimental peri-implantitis and periodontitis when both disease models were initiated at the same time [70, 71]. According to Liu, Liu [72], the higher RANKL/ OPG ratio in peri-implantitis might contribute to the faster rate of bone resorption observed in peri-implantitis progression in comparison to periodontitis, suggesting that the proinflammatory cytokine-mediated bone resorption is relatively more central.

Most of the included studies evaluated the mediators’ levels in PICF. PICF is a serum derivate transude in health or exudate in disease which is located in the peri-implant crevice. It reproduces the degree of inflammatory reaction in peri-implant tissues [49]. According to Casado, Canullo [27], the PICF is in close contact with the bone/implant interface and can reproduce the real immunological events that occur in peri-implant tissue. Noteworthy, in this review, higher IL-1β, IL-6 and RANKL/OPG ratio levels were observed in the PICF of peri-implant mucositis individuals in comparison to healthy individuals. The establishment of an early diagnosis is essential to peri-implantitis prevention since peri-implant mucositis represents the precursor of peri-implantitis [73, 74]. Therefore, the analysis of these modulators in PICF may offer a non-invasive advanced diagnostic method useful for early peri-implant mucositis diagnosis. Further studies focused on these modulators are necessary to confirm these findings. In agreement, lower proinflammatory cytokines (IL-1β and IL-6) and RANKL/OPG ratio were observed in peri-implant mucositis individuals in comparison to peri-implantitis individuals; this could be due to the lower peri-implant mucositis severity compared to peri-implantitis [75].

The main limitations of this review are associated with the quantitative analysis (meta-analysis). Despite the efforts to select high-quality studies comprising with the high comparable aspects possible, high heterogeneity was found between the included studies. The high heterogeneity could be minimized whether there would be studies in the literature with similar criteria to classify an individual as diseased or healthy. Moreover, three studies had their data converted to pg/ml to be included in the meta-analysis. Moreover, unfortunately, few studies evaluating both IL-1β/IL-10, IL-1β/IL-1Ra and IL-6/IL-10 were found in the literature and no studies including the ratio between these cytokines were found. In addition, more studies evaluating these mediators enrolling a larger number of individuals need to be developed to enforce the data shown in the present review.

The challenge for future meta-analyses studies is to find studies designed as similar as possible regarding clinical parameters used for the utilized sampling, selecting patients and the unit of cytokine measurement. Following the new classification of periodontal and peri-implant diseases and conditions published in 2018, the diagnosis of peri-implantitis involves the presence of bleeding and/or suppuration after gentle probing, probing depths of ≥ 6 mm and bone levels ≥ 3 mm apical of the most coronal portion of the intraosseous part of the implant [5].

Summarizing, the present review showed strong evidence that IL-1β, IL-6, IL-10 and RANKL/OPG act in networks in the pathophysiology of peri-implant disease. Increased awareness of peri-implant inflammatory response against microbial infection is important for new therapeutic strategies establishment, as adjuncts for anti-infectious therapies, to modulate the host response [28]. Moreover, the investigation of the inflammatory mediators’ levels has been suggested to detect active sites with peri-implantitis, which may be an instrument for early diagnosis and prevention of this disease [48, 76]. Early diagnosis of peri-implant diseases, mainly the peri-implant mucositis, avoids the need for surgical treatment, thus increasing treatment success with better cost-effectiveness [46].

In conclusion, this systematic review and meta-analysis study showed higher pro-inflammatory (IL-1β, IL-6) and pro-osteoclastogenic (RANKL) levels in PICF of individuals with peri-implant diseases in comparison to healthy individuals. Considering the RANKL/OPG ratio, it was also found a higher level of RANKL and a lower level of OPG in PICF of individuals with peri-implant diseases.

## Supplementary Information

**Additional file 1.** Supplementary materials – Search strategy.

**Additional file 2: Table S1.** Excluded studies with exclusion reasons after full-text assessment.

**Additional file 3: Table S2.** Qualitative analysis of studies which focused on: IL-1β versus IL-10 in peri-implant crevicular fluid; Control versus Mucositis.** Table S3.** Qualitative analysis of studies which focused on: IL-1β versus IL-10 in peri-implant crevicular fluid; Control versus Peri-implantitis.** Table S4.** Qualitative analysis of studies which focused on: IL-1β versus IL-10 in peri-implantar crevicular fluid; Mucositis versus Peri-implantitis.** Table S5.** Qualitative analysis of studies which focused on: IL-1 versus IL-1Ra in saliva; Control versus Peri-implantitis.** Table S6.** Qualitative analysis of studies which focused on: IL-6 versus IL-10 in peri-implant crevicular fluid; Control versus Mucositis.** Table S7.** Qualitative analysis of studies which focused on: IL-6 versus IL-10 in peri-implant crevicular fluid; Control versus Peri-implantitis.** Table S8.** Qualitative analysis of studies which focused on: IL-6 versus IL-10 in peri-implant crevicular fluid; Mucositis versus Peri-implantitis.** Table S9.** Qualitative analysis of studies which focused on: IL-6 versus IL-10 in saliva; Control versus Mucositis.** Table S10.** Qualitative analysis of studies which focused on: IL-6 versus IL-10 in saliva; Control versus Peri-implantitis.** Table S11.** Qualitative analysis of studies which focused on: IL-6 versus IL-10 in saliva; Mucositis versus Peri-implantitis. **Table S12.** Qualitative analysis of studies which focused on: RANKL versus OPG in peri-implant crevicular fluid; Control versus Mucositis.** Table S13.** Qualitative analysis of studies which focused on: RANKL versus OPG in peri-implant crevicular fluid; Control versus Peri-implantitis.** Table S14.** Qualitative analysis of studies which focused on: RANKL versus OPG in peri-implant crevicular fluid; Mucositis versus Peri-implantitis.** Table S15.** Qualitative analysis of studies which focused on: RANKL versus OPG in tissue sample; Control versus Mucositis.** Table S16.** Qualitative analysis of studies which focused on: RANKL versus OPG in tissue sample; Control versus Peri-implantitis.** Table S17.** Qualitative analysis of studies which focused on: RANKL versus OPG in tissue sample; Control versus Peri-implantitis Severe.** Table S18.** Qualitative analysis of studies which focused on: RANKL versus OPG in tissue sample; Mucositis versus Peri-implantitis.** Table S19.** Qualitative analysis of studies which focused on: RANKL versus OPG in tissue sample; Mucositis versus Peri-implantitis Severe.

**Additional file 4: Table S20**. Datas funding and limitations studies.

## Data Availability

The datasets used and/or analyzed during the current study are available from the corresponding author on reasonable request.
