# Supplementary material for: Pro- and anti-inflammatory cytokines and osteoclastogenesis-related factors in peri-implant diseases: systematic review and meta-analysis
Source: BMC Oral Health. 2023 Jun 24;23:420. doi: 10.1186/s12903-023-03072-1 (PMC10290807; doi:10.1186/s12903-023-03072-1)
Supplement: Supplementary file 1 — Additional file 1. Supplementary materials – Search strategy. [file 12903_2023_3072_MOESM1_ESM.docx]

**Supplementary materials – Search strategy**

**2 MATERIALS AND METHODS**

***2.5 Literature search***

In PubMed, Cochrane Controlled Trials Registry, Web of Science and EMBASE databases, the publications were found using the combination of the following terms:

**For the ratio IL-1/IL-10:** “peri-implantitis” OR “periimplantitis” OR “bone resorption” AND “IL-1” OR “interleukin-1” OR “interleukin 1” OR “IL1” OR “interleukin-1alpha” OR “interleukin 1alpha” OR “IL-1 alpha” OR “interleukin 1beta” OR “interleukin-1beta” OR “IL-1 beta” AND “interleukin-10” OR “interleukin 10” OR “IL-10” OR “IL10”;

**For the ratio IL-1/IL-1RA:** “peri-implantitis” OR “periimplantitis” OR “bone resorption” AND “IL-1” OR “interleukin-1” OR “interleukin 1” OR “IL1” OR “interleukin-1alpha” OR “interleukin 1alpha” OR “IL-1 alpha” OR “interleukin 1beta” OR “interleukin-1beta” OR “IL-1 beta” AND “interleukin 1 receptor antagonist protein” OR “IL-1Ra” OR “interleukin 1 receptor blocking agent” OR “IL1RN”;

**For the ratio IL-6/IL-10:** “peri-implantitis” OR “periimplantitis” OR “bone resorption” AND “IL-6” OR “interleukin-6” OR “interleukin 6” OR “IL6” AND “interleukin-10” OR “IL-10” OR “interleukin 10” OR “IL10”; and

**For the ratio RANKL/OPG:** peri-implantitis” OR “periimplantitis” AND "tnfrsf11" OR "tumor necrosis factor ligand superfamily member 11" OR "TNF superfamily member 11" OR "receptor activator of nuclear factor-kappa B ligand" OR "RANKL" OR “osteoclast differentiation factor” AND "tnfrsf11b" OR "tumor necrosis factor receptor superfamily member 11b" OR "osteoprotegerin” OR “OPG”.

In Scopus database, the publications were found using the combination of the following terms:

**For the ratio IL-1/IL-10:** “peri-implantitis” OR “periimplantitis” AND “IL-1” OR “interleukin-1” OR “interleukin 1” OR “IL1” OR “interleukin-1alpha” OR “interleukin 1alpha” OR “IL-1 alpha” OR “interleukin 1beta” OR “interleukin-1beta” OR “IL-1 beta” AND “interleukin-10” OR “interleukin 10” OR “IL-10” OR “IL10”;

**For the ratio IL-1/IL-1RA:** “peri-implantitis” OR “periimplantitis” AND “IL-1” OR “interleukin-1” OR “interleukin 1” OR “IL1” OR “interleukin-1alpha” OR “interleukin 1alpha” OR “IL-1 alpha” OR “interleukin 1beta” OR “interleukin-1beta” OR “IL-1 beta” AND “interleukin 1 receptor antagonist protein” OR “IL-1Ra” OR “interleukin 1 receptor blocking agent” OR “IL1RN”;

**For the ratio IL-6/IL-10:** “peri-implantitis” OR “periimplantitis” AND “IL-6” OR “interleukin-6” OR “interleukin 6” OR “IL6” AND “interleukin-10” OR “IL-10” OR “interleukin 10” OR “IL10”; and

**For the ratio RANKL/OPG:** “peri-implantitis” OR “periimplantitis” AND "tnfrsf11" OR "tumor necrosis factor ligand superfamily member 11" OR "TNF superfamily member 11" OR "receptor activator of nuclear factor-kappa B ligand" OR "RANKL" OR “osteoclast differentiation factor” AND "tnfrsf11b" OR "tumor necrosis factor receptor superfamily member 11b" OR "osteoprotegerin” OR “OPG”.

In Google Scholar database (gray literature), the publications were found using the combination of the following terms:

**For the ratio IL-1/IL-10:** “peri-implantitis” AND “Interleukin 1” AND “Interleukin 10” AND “Clinical study”

**For the ratio IL-1/IL-1RA:** “peri-implantitis” AND “Interleukin 1” AND “Interleukin 1Ra” AND “Clinical study”

**For the ratio IL-6/IL-10:** “peri-implantitis” AND “Interleukin 1” AND “Interleukin 6” AND “Clinical study”

**For the ratio RANKL/OPG:** “peri-implantitis” AND “RANKL” AND “OPG” AND “Clinical study”
