# Supplementary material for: Pro- and anti-inflammatory cytokines and osteoclastogenesis-related factors in peri-implant diseases: systematic review and meta-analysis
Source: BMC Oral Health. 2023 Jun 24;23:420. doi: 10.1186/s12903-023-03072-1 (PMC10290807; doi:10.1186/s12903-023-03072-1)
Supplement: Supplementary file 2 — Additional file 2: Table S1. Excluded studies with exclusion reasons after full-text assessment. [file 12903_2023_3072_MOESM2_ESM.docx]

| **Author/Year** | **Exclusion reasons** |
| --- | --- |
| Abbas, Clohisy (1) | In vitro study. |
| Cionca, Hashim (2) | Control group absence. |
| Esberg, Isehed (3) | Control group absence; investigated only IL-1. |
| Gleiznys, Gleiznys (4) | In vitro study. |
| Gomes, de Oliveira (5) | Patient with and without regular maintenance therapy. |
| Kadkhodazadeh, Tabari (6) | Polymorphism study; investigated only OGP. |
| Konermann, Götz (7) | Histochemistry and immunohistochemistry study. |
| Laine, Leonhardt (8) | Polymorphism study. |
| Li, Qi (9) | In vitro and in vivo study. |
| Liu, Liu (10) | Control group absence. |
| Monov, Strbac (11) | Control and disease group absence. |
| Montes, Alvim-Pereira (12) | Polymorphism study. |
| Onuma, Aquiar (13) | Peri-implantitis disease group absence. |
| Öztürk, Emingil (14) | Peri-implantitis disease group absence. |
| Peker Tekdal, Bostanci (15) | Peri-implantitis disease group absence. |
| Rakic, Nikolic-Jakoba (16) | Investigated only RANK. |
| Ribeiro, Melo (17) | Polymorphism study. |
| Ribeiro, Casati (18) | Experimental peri-implant mucositis study model. |
| Sampaio-Fernandes, Vaz (19) | Polymorphism study. |
| Sampaio-Fernandes, Vaz (20) | Polymorphism study. |
| Sarlati, Sattari (21) | Investigated only RANKL. |
| Zhou and Zhao (22) | Polymorphism study. |
| Zhou, Lin (23) | In vitro study. |
| Hentenaar, De Waal (24) | No data about RANKL. |
| Karci and Oncu (25) | Comparison between two different implant types. |
| Liskmann, Vihalemm (26) | Groups divided by probing depth. |
| Negri, Pimentel (27) | Control and disease group absence. |
| Nowzari, Botero (28) | Peri-implantitis disease group absence. |
| Saremi, Shafizadeh (29) | Polymorphism study. |
| Djinic Krasavcevic, Nikolic (30) | Control and disease group absence. |
| Silva, Reis (31) | Polymorphism study. |

**Table S1:** Excluded studies with exclusion reasons after full-text assessment.

**REFERENCES TABLE S1**

1. Abbas S, Clohisy JC, Abu-Amer Y. Mitogen-activated protein (MAP) kinases mediate PMMA-induction of osteoclasts. Journal of Orthopaedic Research. 2003;21(6):1041-8.

2. Cionca N, Hashim D, Cancela J, Giannopoulou C, Mombelli A. Pro-inflammatory cytokines at zirconia implants and teeth. A cross-sectional assessment. Clinical oral investigations. 2016;20(8):2285-91.

3. Esberg A, Isehed C, Holmlund A, Lundberg P. Peri-implant crevicular fluid proteome before and after adjunctive enamel matrix derivative treatment of peri-implantitis. Journal of clinical periodontology. 2019;46(6):669-77.

4. Gleiznys D, Gleiznys A, Abraskeviciute L, Vitkauskiene A, Saferis V, Sakalauskiene J. Interleukin-10 and Interleukin-1beta Cytokines Expression in Leukocytes of Patients with Chronic Peri-Mucositis. Medical science monitor : international medical journal of experimental and clinical research. 2019;25:7471-9.

5. Gomes AM, de Oliveira DWG, Ferreira SD, Silva TA, Cota LOM, Costa FO. Periodontal disease, peri-implant disease and levels of salivary biomarkers IL-1β, IL-10, RANK, OPG, MMP-2, TGF-β and TNF-α: follow-up over 5 years. Journal of applied oral science : revista FOB. 2019;27:e20180316‐.

6. Kadkhodazadeh M, Tabari ZA, Ardakani MRT, Ebadian AR, Brook A. Analysis of osteoprotegerin (OPG) gene polymorphism in Iranian patients with chronic periodontitis and peri-implantitis. A cross-sectional study. European journal of oral implantology. 2012;5(4):381-8.

7. Konermann A, Götz W, Le M, Dirk C, Lossdörfer S, Heinemann F. Histopathological Verification of Osteoimmunological Mediators in Peri-Implantitis and Correlation to Bone Loss and Implant Functional Period. The Journal of oral implantology. 2016;42(1):61-8.

8. Laine ML, Leonhardt Å, Roos-Jansåker AM, Peña AS, Van Winkelhoff AJ, Winkel EG, et al. IL-1RN gene polymorphism is associated with peri-implantitis. Clinical oral implants research. 2006;17(4):380-5.

9. Li X, Qi ML, Sun XL, Weir MD, Tay FR, Oates TW, et al. Surface treatments on titanium implants via nanostructured ceria for antibacterial and anti-inflammatory capabilities. Acta biomaterialia. 2019;94:627-43.

10. Liu YD, Liu QF, Li ZP, Acharya A, Chen DY, Chen ZT, et al. Long non-coding RNA and mRNA expression profiles in peri-implantitis vs periodontitis. Journal of periodontal research.

11. Monov G, Strbac GD, Baron M, Kandler B, Watzek G, Gruber R. Soluble RANKL in crevicular fluid of dental implants: A pilot study. Clinical implant dentistry and related research. 2006;8(3):135-41.

12. Montes CC, Alvim-Pereira F, de Castilhos BB, Sakurai MLL, Olandoski M, Trevilatto PC. Analysis of the association of IL1B (C+3954T) and IL1RN (intron 2) polymorphisms with dental implant loss in a Brazilian population. Clinical oral implants research. 2009;20(2):208-17.

13. Onuma T, Aquiar K, Duarte PM, Feres M, Giro G, Coelho P, et al. Levels of osteoclastogenesis-related factors in the peri-implant crevicular fluid and clinical parameters of immediately loaded implants in patients with osteopenia: a short-term report. International journal of oral & maxillofacial implants. 2015;30(6):1431‐6.

14. Öztürk V, Emingil G, Bostanci N, Belibasakis GN. Impact of implant-abutment connection on osteoimmunological and microbiological parameters in short implants: a randomized controlled clinical trial. Clinical oral implants research. 2017;28(9):e111‐e20.

15. Peker Tekdal G, Bostanci N, Belibasakis GN, Gürkan A. The effect of piezoelectric surgery implant osteotomy on radiological and molecular parameters of peri-implant crestal bone loss: a randomized, controlled, split-mouth trial. Clinical oral implants research. 2016;27(5):535‐44.

16. Rakic M, Nikolic-Jakoba N, Struillou X, Petkovic-Curcin A, Stamatovic N, Matic S, et al. Receptor activator of nuclear factor kappa B (RANK) as a determinant of peri-implantitis. Vojnosanitetski Pregled. 2013;70(4):346-51.

17. Ribeiro R, Melo R, Neto PT, Vajgel A, Souza PRE, Cimoes R. Polymorphisms of Il-10(-1082) and RANKL (-438) Genes and the Failure of Dental Implants. International Journal of Dentistry. 2017.

18. Ribeiro FV, Casati MZ, Casarin RC, Correa MG, Cirano FR, Negri BM, et al. Impact of a triclosan-containing toothpaste during the progression of experimental peri-implant mucositis: Clinical parameters and local pattern of osteo-immunoinflammatory mediators in peri-implant fluid. Journal of periodontology. 2018;89(2):203-12.

19. Sampaio-Fernandes M, Vaz P, Fonseca P, Reis-Campos J, Figueiral MH. IL1 gene cluster polymorphisms and peri-implant disease. Jorge RMN, Campos JCR, Vaz MAP, Santos SM, Tavares J, editors2014. 197-9 p.

20. Sampaio-Fernandes M, Vaz PC, Braga AC, Figueiral MH. IL1RN gene polymorphism in a Portuguese population with implant-supported overdentures - An observational study. Revista Portuguesa de Estomatologia, Medicina Dentaria e Cirurgia Maxilofacial. 2015;56(4):207-14.

21. Sarlati F, Sattari M, Gazar AG, Rafsenjani AN. Receptor Activator of Nuclear Factor Kappa B Ligand (RANKL) Levels in Peri-Implant Crevicular Fluid. Iranian Journal of Immunology. 2010;7(4):226-33.

22. Zhou J, Zhao YM. Osteoprotegerin Gene (OPG) Polymorphisms Associated with Peri-Implantitis Susceptibility in a Chinese Han Population. Medical Science Monitor. 2016;22:4271-6.

23. Zhou L, Lin Z, Ding J, Huang W, Chen J, Wu D. Inflammatory and biocompatibility evaluation of antimicrobial peptide GL13K immobilized onto titanium by silanization. Colloids and Surfaces B: Biointerfaces. 2017;160:581-8.

24. Hentenaar DFM, De Waal YCM, Vissink A, Van Winkelhoff AJ, Meijer HJA, Liefers SC, et al. Biomarker levels in peri-implant crevicular fluid of healthy implants, untreated and non-surgically treated implants with peri-implantitis. Journal of clinical periodontology. 2021;48(4):590-601.

25. Karci B, Oncu E. Comparison of osteoimmunological and microbiological parameters of extra short and longer implants loaded in the posterior mandible: a split mouth randomized clinical study. Acta stomatologica Croatica. 2021;55(3):238‐47.

26. Liskmann S, Vihalemm T, Salum O, Zilmer K, Fischer K, Zilmer M. Correlations between clinical parameters and interleukin-6 and interleukin-10 levels in saliva from totally edentulous patients with peri-implant disease. Int J Oral Maxillofac Implants. 2006;21(4):543-50.

27. Negri BM, Pimentel SP, Casati MZ, Cirano FR, Casarin RC, Ribeiro FV. Impact of a chronic smoking habit on the osteo-immunoinflammatory mediators in the peri-implant fluid of clinically healthy dental implants. Arch Oral Biol. 2016;70:55-61.

28. Nowzari H, Botero JE, DeGiacomo M, Villacres MC, Rich SK. Microbiology and cytokine levels around healthy dental implants and teeth. Clin Implant Dent Relat Res. 2008;10(3):166-73.

29. Saremi L, Shafizadeh M, Esmaeilzadeh E, Ghaffari ME, Mahdavi M, Amid R, et al. Assessment of IL-10, IL-1ß and TNF-α gene polymorphisms in patients with peri-implantitis and healthy controls. Molecular biology reports. 2021;48(3):2285-90.

30. Djinic Krasavcevic A, Nikolic N, Milinkovic I, Carkic J, Jezdic M, Jankovic S, et al. Notch signalling cascade and proinflammatory mediators in peri-implant lesions with different RANKL/OPG ratios-An observational study. Journal of periodontal research. 2023.

31. Silva RC, Reis MBL, Arid J, Flores EKB, Cruz GV, Marañón-Vásquez GA, et al. Association between genetic polymorphisms in rank, rankl and opg and peri-implant diseases in patients from the amazon region. Brazilian Dental Journal. 2020;31(1):63-8.
