# Supplementary material for: Pro- and anti-inflammatory cytokines and osteoclastogenesis-related factors in peri-implant diseases: systematic review and meta-analysis
Source: BMC Oral Health. 2023 Jun 24;23:420. doi: 10.1186/s12903-023-03072-1 (PMC10290807; doi:10.1186/s12903-023-03072-1)
Supplement: Supplementary file 3 — Additional file 3: Table S2. Qualitative analysis of studies which focused on: IL-1β versus IL-10 in peri-implant crevicular fluid; Control versus Mucositis. Table S3. Qualitative analysis of studies which focused on: IL-1β versus IL-10 in peri-implant crevicular fluid; Control versus Peri-implantitis. Table S4. Qualitative analysis of studies which focused on: IL-1β versus IL-10 in peri-implantar crevicular fluid; Mucositis versus Peri-implantitis. Table S5. Qualitative analysis of studies which focused on: IL-1 versus IL-1Ra in saliva; Control versus Peri-implantitis. Table S6. Qualitative analysis of studies which focused on: IL-6 versus IL-10 in peri-implant crevicular fluid; Control versus Mucositis. Table S7. Qualitative analysis of studies which focused on: IL-6 versus IL-10 in peri-implant crevicular fluid; Control versus Peri-implantitis. Table S8. Qualitative analysis of studies which focused on: IL-6 versus IL-10 in peri-implant crevicular fluid; Mucositis versus Peri-implantitis. Table S9. Qualitative analysis of studies which focused on: IL-6 versus IL-10 in saliva; Control versus Mucositis. Table S10. Qualitative analysis of studies which focused on: IL-6 versus IL-10 in saliva; Control versus Peri-implantitis. Table S11. Qualitative analysis of studies which focused on: IL-6 versus IL-10 in saliva; Mucositis versus Peri-implantitis. Table S12. Qualitative analysis of studies which focused on: RANKL versus OPG in peri-implant crevicular fluid; Control versus Mucositis. Table S13. Qualitative analysis of studies which focused on: RANKL versus OPG in peri-implant crevicular fluid; Control versus Peri-implantitis. Table S14. Qualitative analysis of studies which focused on: RANKL versus OPG in peri-implant crevicular fluid; Mucositis versus Peri-implantitis. Table S15. Qualitative analysis of studies which focused on: RANKL versus OPG in tissue sample; Control versus Mucositis. Table S16. Qualitative analysis of studies which focused on: RANKL versus OPG [file 12903_2023_3072_MOESM3_ESM.docx]

**SUPPLEMENTARY MATERIAL**

**Pro- and anti-inflammatory cytokines and osteoclastogenesis-related factors in peri-implant diseases: systematic review and meta-analysis**

Tables S1-S23 (supplementary materials) support the systematic review which utilized qualitative analysis. Data from each study is summarized presented, intending to show the levels of pro- and anti-inflammatory cytokines and osteoclastogenesis-related factors in tissue samples (gene expression) and biofluids (protein measurement). Because the methodologies to assess protein measurement are different, these tables did not intend to compare the methods, but just to widely present the levels of the peri-implantitis modulators investigated.

| **Table S2:** Qualitative analysis of studies which focused on: IL-1β versus IL-10 in peri-implant crevicular fluid; Control versus Mucositis | | | | | | | | |
| --- | --- | --- | --- | --- | --- | --- | --- | --- |
| **IL-1β versus IL-10 – Peri-implant Crevicular Fluid - Control versus Mucositis** | | | | | | | | |
|  | | Mean age (year) | | Diagnostic Criteria | |  | | |
| Author Year | Sample Size | Control | Disease | Control | Disease | Evaluation Method | IL-1β  Levels | IL-10  Levels |
| Casado, Canullo (1) | CG: 10  DG: 10 | 49.5 | 52.8 | No clinical signs of inflammation in the peri-implant mucosa and no sign of bone loss in all regions. | Implants with BOP, red mucosa and swelling, spontaneous bleeding, but no radiographic signs of pathologic bone loss. | ELISA | HIDS^#^ | LIDS* |
| Guncu, Akman (2) | CG:20  DG:27 | NI | NI | Implants with Gingival Index = 0 | Implantts with Gingival Index > 0 | PCR-RT | HIDS* | HIDS* |
| Kandaswamy, Sakulpaptong (3) | CG: 25 implants  DG: 33 implants | NI | NI | Absence of visual signs of inflammation and bleeding on probing. | Bleeding on probing and visual signs of inflammation. | Multiplex | HIDS* | HIDS* |

CG: Control Group; DG: Disease Group; NI: Not informed; IL-: Interleukin; BOP: Bleeding On Probing; HIDS: Higher In Diseased Subjects; LIDS: Lower In Diseased Subjects. *p˂0.05; #p<0.0005.

| **Table S3:** Qualitative analysis of studies which focused on: IL-1β versus IL-10 in peri-implant crevicular fluid; Control versus Peri-implantitis | | | | | | | | |
| --- | --- | --- | --- | --- | --- | --- | --- | --- |
| **IL-1β versus IL-10 – Peri-implant Crevicular Fluid - Control versus Peri-implantitis** | | | | | | | | |
|  | | Mean age (year) | | Diagnostic Criteria | |  | | |
| Author Year | Sample Size | Control | Disease | Control | Disease | Evaluation Method | IL-1β  Levels | IL-10  Levels |
| Ata-Ali, Flichy-Fernandez (4) | CG: 54 DG: 24 | 63.6 | 52 | PD < 4 mm, absence of clinical signs of inflammation of the peri-implant mucosa, and without radiographic bone loss. | Implant with a PD =4 mm and signs of acute  peri-implantitis (loss of supporting bone as estimated on radiographs, BOP, or suppuration) and no implant mobility. | Multiplex | HIDS** | HIDS* |
| Casado, Canullo (1) | CG: 10  DG: 10 | 49.5 | 57.4 | No clinical signs of inflammation in the peri-implant mucosa and no sign of bone loss in all regions. | Clinical signs of inflammation, including implant mobility and suppuration in some cases, and radiographic signs of bone loss. | ELISA | HIDS*** | LIDS**** |
| Ghighi, Llorens (5) | CG:10  DG:11 | NI | NI | Patients underwent surgery for erupted third molar extraction and should not have either history of periodontitis or peri-implantitis according to clinical criteria of gingival bleeding, PD and radiographic evidence of bone resorptions. | Patients should present at least one dental implant in function with a titanium abutment, a PD≥5mm with BOP and radiographic evidence of bone loss. | Multiplex | HIDS** | HIDS* |
| Kandaswamy, Sakulpaptong (3) | CG: 25 implants  DG: 59 implants | NI | NI | Absence of visual signs of inflammation and bleeding on probing. | Presence of inflammation in the peri-implant mucosa and subsequent progressive loss of supporting bone. | Multiplex | HIDS* | HIDS* |

CG: control group; DG: Disease Group; NI: Not informed; IL-: Interleukin; HIDS: Higher in Diseased Subjects; LIDS: Lower In Diseased Subjects. *p<0.05; **p<0.01; ***p ˂ 0.001; ****p<0.0001.

| **Table S4:** Qualitative analysis of studies which focused on: IL-1β versus IL-10 in peri-implantar crevicular fluid; Mucositis versus Peri-implantitis | | | | | | | | |
| --- | --- | --- | --- | --- | --- | --- | --- | --- |
| **IL-1β versus IL-10 – Peri-implant Crevicular Fluid - Mucositis versus Peri-implantitis** | | | | | | | | |
|  | | Mean age (year) | | Diagnostic Criteria | |  | | |
| Author Year | Sample Size | M | PI | M | PI | Evaluation Method | IL-1β  Levels | IL-10  Levels |
| Casado, Canullo (1) | M: 10  PI: 10 | 52.8 | 57.4 | Implants with BOP, red mucosa and swelling, spontaneous bleeding, but no radiographic signs of pathologic bone loss. | Clinical signs of inflammation, including implant mobility and suppuration in some cases, and radiographic signs of bone loss. | ELISA | HIPS | LIPS* |
| Fonseca, Moraes Junior (6) | M: 12  PI: 10 | 65 | 59.4 | Patients who showed inflamed sites with bone loss around the implants no deeper than the first implant’s thread and PD ≤3 mm. | Patients who showed inflamed sites with at least one implant with bone loss around two or more threads of the implant and pocket depth ≥4 mm. | Multiplex | HIPS | LIPS |
| Kandaswamy, Sakulpaptong (3) | M: 33 implants  PI: 59 implants | NI | NI | Bleeding on probing and visual signs of inflammation. | Presence of inflammation in the peri-implant mucosa and subsequent progressive loss of supporting bone. | Multiplex | HIPS | LIPS |
| Teixeira, Lira‐Junior (7) | M: 10  PI: 14 | 59.8 | 59.9 | Clinically inflamed sites and no significant radiographic bone loss | Inflamed sites and bone loss involving two or more implant threads. | Multiplex | LIPS | LIPS |

M: Mucositis; PI: Peri-implantitis; NI: Not informed; BOP: Bleeding on Probing; IL-: Interleukin; HIPS: Higher In Peri-implantitis Subjects; LIPS: Lower In Peri-implantitis Subjects. *p<0.05.

| **Table S5:** Qualitative analysis of studies which focused on: IL-1 versus IL-1Ra in saliva; Control versus Peri-implantitis | | | | | | | | |
| --- | --- | --- | --- | --- | --- | --- | --- | --- |
| **IL-1β versus IL-1Ra – Peri-implant Crevicular Fluid - Control versus Peri-implantitis** | | | | | | | | |
|  | | Mean age (year) | | Diagnostic Criteria | |  | | |
| Author Year | Sample Size | CG | PI | CG | PI | Evaluation Method | IL-1β  Levels | IL-1Ra  Levels |
| Song, Jiang (8) | CG: 14 implants  DG: 14 implants | NI | NI | With an absence of soft tissue inflammation and further additional bone loss following initial healing according to radiographic examination at baseline and at follow-up | With bone loss and increasing PD following initial healing, or with MBL ≥ 3 mm and PD ≥ 6 mm without previous examination data. | Multiplex | HIDS*** | LIDS |

CG: Control Group; PI: Peri-implantitis; NI: Not informed; IL-: Interleukin; PD: Probing Depth; HIDS: Higher In Diseased Subjects; LIDS: Lower In Disease Subjects. ***p ˂ 0.001.

| **Table S6:** Qualitative analysis of studies which focused on: IL-6 versus IL-10 in peri-implant crevicular fluid; Control versus Mucositis | | | | | | | | |
| --- | --- | --- | --- | --- | --- | --- | --- | --- |
| **IL-6 versus IL-10 – Peri-implant Crevicular Fluid - Control versus Mucositis** | | | | | | | | |
|  | | Mean age (year) | | Diagnostic Criteria | |  | | |
| Author Year | Sample Size | Control | Disease | Control | Disease | Evaluation Method | IL-6  Levels | IL-10  Levels |
| Kandaswamy, Sakulpaptong (3) | CG: 25 implants  DG: 33 implants | NI | NI | Absence of visual signs of inflammation and bleeding on probing. | Bleeding on probing and visual signs of inflammation. | Multiplex | HIDS** | HIDS* |
| Severino, Beghini (9) | CG: 10  DG: 20 | 75.20 | 60.88 | Implants with PD of 0–3 mm, without marginal bleeding, suppuration, or bone loss. | Implants with PD of 0–3 mm, with marginal bleeding, without suppuration or bone loss. | ELISA | HIDS | HIDS |

CG: Control Group; DG: Disease Group; NI: Not informed; IL-: Interleukin; PD: Probing Depth; HIDS: Higher In Diseased Subjects; *p<0.05;

**p<0.01.

| **Table S7:** Qualitative analysis of studies which focused on: IL-6 versus IL-10 in peri-implant crevicular fluid; Control versus Peri-implantitis | | | | | | | | | |
| --- | --- | --- | --- | --- | --- | --- | --- | --- | --- |
| **IL-6 versus IL-10 – Peri-implant Crevicular Fluid - Control versus Peri-implantitis** | | | | | | | | | |
|  | | Mean age (year) | | Diagnostic Criteria | |  | | | |
| Author Year | Sample Size | Control | Disease | Control | Disease | Evaluation Method | IL-6  Levels | IL-10  Levels | |
| Ata-Ali, Flichy-Fernandez (4) | CG: 54 DG: 24 | 63,6 | 52 | PD < 4 mm, absence of clinical signs of inflammation of the peri-implant mucosa, and without radiographic bone loss. | Implant with a PD =4 mm and signs of acute  peri-implantitis (loss of supporting bone as estimated on radiographs, BOP or suppuration) and no implant mobility. | Multiplex | HIDS** | HIDS* |  |
| Fonseca, Moraes Junior (6) | DG1: 12  DG2: 10 | 65 | 59,4 | Patients who showed inflamed sites with bone loss around the implants no deeper than the first implant’s thread and PD ≤3 mm. | Patients who showed inflamed sites with at least one implant with bone loss around two or more threads of the implant and pocket depth ≥4 mm. | Multiplex | HIDS | LIDS |  |
| Kandaswamy, Sakulpaptong (3) | CG: 25 implants  DG: 59 implants | NI | NI | Absence of visual signs of inflammation and bleeding on probing. | Presence of inflammation in the peri-implant mucosa and subsequent progressive loss of supporting bone. | Multiplex | HIDS** | HIDS* |  |
| Severino, Napimoga (10) | CG: 20 implants  DG: 20 implants | 52.27 | 47.5 | Implants with PD of 0–2 mm without MB, suppuration or bone loss. | PD greater than or equal to 3 mm in at least one site of the implant and MB. Implants with 1-wall or horizontal bone defects | ELISA | HIDS | LIDS | |
| Severino, Beghini (9) | CG: 10  DG: 20 | 75.20 | 60.81 | Implants with probing depth of 0–3 mm, without marginal bleeding, suppuration, or bone loss. | Implants with probing depth of 0–3 mm, with marginal bleeding, without suppuration or bone loss. | ELISA | HIDS** | HIDS | |

CG: Control Group; DG: Disease Group; NI: Not informed; IL-: Interleukin; PD: Probing Depth; MB: Marginal bleeding; HIDS: Higher In Diseased Subjects; LIDS: Lower In Disease Subjects. *p<0.05; **p<0.01.

| **Table S8:** Qualitative analysis of studies which focused on: IL-6 versus IL-10 in peri-implant crevicular fluid; Mucositis versus Peri-implantitis | | | | | | | | |
| --- | --- | --- | --- | --- | --- | --- | --- | --- |
| **IL-6 versus IL-10 – Peri-implant Crevicular Fluid - Mucositis versus Peri-implantitis** | | | | | | | | |
|  | | Mean age (year) | | Diagnostic Criteria | |  | | |
| Author Year | Sample Size | M | PI | M | PI | Evaluation Method | IL-6  Levels | IL-10  Levels |
| Kandaswamy, Sakulpaptong (3) | M: 33 implants  PI: 59 implants | NI | NI | Bleeding on probing and visual signs of inflammation. | Presence of inflammation in the peri-implant mucosa and subsequent progressive loss of supporting bone. | Multiplex | LIPS* | LIPS |
| Severino, Beghini (9) | M: 10  PI: 20 | 60.88 | 60.81 | Implants with PD of 0–3 mm, without marginal bleeding, suppuration, or bone loss. | Implants with PD of 0–3 mm, with marginal bleeding, without suppuration or bone loss. | ELISA | HIPS | LIPS |
| Teixeira, Lira‐Junior (7) | M: 10  PI: 14 | 59.8 | 59.9 | Clinically inflamed sites and no significant radiographic bone loss. | Inflamed sites and bone loss involving two or more implant threads. | Multiplex | LIPS | LIPS |

M: Mucositis; PI: Peri-Implantitis; NI: Not informed; IL-: Interleukin; PD: Probing Depth; HIPS: Higher In Peri-implantitis Subjects; LIPS: Lower In Peri-implantitis Subjects; *p<0.05.

| **Table S9:** Qualitative analysis of studies which focused on: IL-6 versus IL-10 in saliva; Control versus Mucositis | | | | | | | | | |
| --- | --- | --- | --- | --- | --- | --- | --- | --- | --- |
| **IL-6 versus IL-10 – Saliva - Control versus Mucositis** | | | | | | | | | |
|  | | Mean age (year) | | Diagnostic Criteria | |  | | | |
| Author Year | Sample Size | Control | Disease | Control | Disease | Evaluation Method | IL-6  Levels | IL-10  Levels |  |
| Severino, Beghini (9) | CG: 10  DG: 20 | 75.20 | 60.88 | Implants with PD of 0–3 mm, without marginal bleeding, suppuration, or bone loss. | Implants with PD of 0–3 mm, with marginal bleeding, without suppuration or bone loss. | ELISA | HIDS | HIDS |  |

CG: Control Group; DG: Disease Group; IL-: Interleukin; PD: Probing Depth; HIDS: Higher In Diseased Subjects.

| **Table S10:** Qualitative analysis of studies which focused on: IL-6 versus IL-10 in saliva; Control versus Peri-implantitis | | | | | | | | |
| --- | --- | --- | --- | --- | --- | --- | --- | --- |
| **IL-6 versus IL-10 – Saliva - Control versus Peri-implantitis** | | | | | | | | |
|  | | Mean age (year) | | Diagnostic Criteria | |  | | |
| Author Year | Sample Size | Control | Disease | Control | Disease | Evaluation Method | IL-6  Levels | IL-10  Levels |
| Severino, Beghini (9) | CG: 10  DG: 20 | 75.20 | 60.81 | Implants with PD of 0–3 mm, without marginal bleeding, suppuration, or bone loss. | Implants with marginal bleeding, with PD greater than 3 mm and bone loss in at least one site of implant | ELISA | HIDS | LIDS |

CG: Control Group; DG: Disease Group; IL-: Interleukin; PD: Probing Depth; HIDS: Higher in Disease Subjects; LIDS: Lower in Disease Subjects.

| **Table S11:** Qualitative analysis of studies which focused on: IL-6 versus IL-10 in saliva; Mucositis versus Peri-implantitis | | | | | | | | |
| --- | --- | --- | --- | --- | --- | --- | --- | --- |
| **IL-6 versus IL-10 – Saliva - Mucositis versus Peri-implantitis** | | | | | | | | |
|  | | Mean age (year) | | Diagnostic Criteria | |  | | |
| Author Year | Sample Size | M | PI | M | PI | Evaluation Method | IL-6  Levels | IL-10  Levels |
| Severino, Beghini (9) | M: 10  PI: 20 | 60.88 | 60.81 | Implants with PD of 0–3 mm, with marginal bleeding, without suppuration or bone loss. | Implants with marginal bleeding, with PD greater than 3 mm and bone loss in at least one site of implant | ELISA | HIPS | LIPS |

M: Mucositis; PI: Peri-implantitis; IL-: Interleukin; PD: Probing Depth; HIPS: Higher In Peri-implantitis Subjects; LIPS: Lower In Peri-implantitis.

| T**able S12:** Qualitative analysis of studies which focused on: RANKL versus OPG in peri-implant crevicular fluid; Control versus Mucositis | | | | | | | | |
| --- | --- | --- | --- | --- | --- | --- | --- | --- |
| **RANKL versus OPG – Peri-implant Crevicular Fluid - Control versus Mucositis** | | | | | | | | |
|  | | Mean age (year) | | Diagnostic Criteria | |  | | |
| Author Year | Sample Size | Control | Disease | Control | Disease | Evaluation Method | RANKL  Levels | OPG  Levels |
| Arıkan, Buduneli (11) | CG: 79 DG: 4 | 53.5 | 52.8 | No PD deeper than 4mm, no suppuration, no plaque or gingival inflammation, and indicated no sign of bone loss in the radiographs. | Implants with BOP, no suppuration or radiographic evidence of bone loss and no PD deeper than 5mm. | ELISA | LIDS | HIDS |
| Chaparro, 2020 | CG: 17  DG: 19 | NI | NI | Absence of swelling, bleeding on probing, inflammation and suppuration; besides the absence of increased probing depth and the absence of radiographic bone loss | Inflammation of the peri-implant soft tissues, without bone loss, but with bleeding on probing, swelling, and suppuration in some cases | Multiplex | LIDS | HIDS |
| Chaparro, Beltran (12) | CG: 7  DG: 2 | 73.8 | 61.8 | Absence of visual signs of inflammation and bleeding on probing. | Bleeding on probing and visual signs of inflammation. | Multiplex | HIDS | HIDS |
| Duarte, de Mendonça (13) | CG: 10 DG:10 | 49.1 | 55.8 | No marginal bleeding, bleeding on probing, suppuration and radiographic bone loss. | Implants with marginal bleeding and/or bleeding on probing, and absence of radiographic bone loss and suppuration. | ELISA | HIDS | LIDS |
| Guncu, Akman (2) | CG:20  DG:27 | NI | NI | Implants with Gingival Index = 0 | Implantts with Gingival Index > 0 | PCR-RT | HIDS | HIDS* |
| Milinkovic, Djinic Krasavcevic (14) | CG: 35  DG:45 | 41,57 | 45,98 | Absence of clinical signs of inflammation; absence of bleeding and/or suppuration on gentle probing; no increase in PD compared to previous examination; absence of bone loss beyond crestal bone level changes resulting from initial bone remodeling. | Bleeding and/or suppuration on gentle probing with or without increased PD compared to previous examinations; absence of continuing bone loss as observed on a radiograph: absence  of loss beyond crestal bone level changes resulting from initial bone remodeling. | ELISA | HIDS | HIDS |
| Rakic, Struillou (15) | CG: 58  DG: 52 | 54,66 | 51,83 | These controls were defined by PD≤3mm, no BOP, and no bone loss evidence by radiograph. | Cases were defined by the presence of peri-implant PD≥3mm, with positive BOP and absence of radiographic bone loss compared with the radiograph taken the time of prosthetic replacement. | ELISA | HIDS | LIDS |
| Rakic, Monje (16) | CG: 126  DG:57 | - | 52.5 | Implants with negative BOP or BOP positive in 1/6 sites being considered the consequence of trauma, with PD<3mm and without evidence of radiological bone loss. | Implants with BOP>0.16, positive in>1 point, PD>3mm and radiological bone loss <2mm | ELISA | HIDS*** | HIDS*** |

CG: Control Group; DG: Disease Group; NI: Not informed; RANKL: Receptor Activator of Nuclear Factor Kappa-B Ligand; OPG: Osteoprotegerin; PD: Probing Depth; BOP: Bleeding On Probing; HIDS: Higher In Diseased Subjects; LIDS: Lower In Diseased Subjects. ***p ˂ 0.001.

| **Table S13:** Qualitative analysis of studies which focused on: RANKL versus OPG in peri-implant crevicular fluid; Control versus Peri-implantitis | | | | | | | | |
| --- | --- | --- | --- | --- | --- | --- | --- | --- |
| **RANKL versus OPG – Peri-implant Crevicular Fluid - Control versus Peri-implantitis** | | | | | | | | |
|  | | Mean age (year) | | Diagnostic Criteria | |  | | |
| Author Year | Sample Size | Control | Disease | Control | Disease | Evaluation Method | RANKL  Levels | OPG  Levels |
| Arıkan, Buduneli (11) | CG: 79 DG: 3 | 53.5 | 66.3 | No PDs deeper than 4mm, no suppuration, no plaque or gingival inflammation, and indicated no sign of bone loss in the radiographs. | Implants with PD deeper than 5mm, BOP, suppuration and radiographic evidence of crestal bone loss in at least one site. | ELISA | LIDS | HIDS |
| Arikan, Buduneli (17) | CG: 21  DG: 18 | 52 | 56 | Absence of PD deeper than 4 mm, an absence of suppuration, absence of plaque, absence of gingival inflammation in terms of BOP, and a lack of radiographic signs of bone loss. | Implants with the PD of at least one measurement site  and frequently more than one-was = 5  mm with BOP and/or suppuration. Radiographic evidence of crestal bone loss involving  at least three threads in at least one site but no more  than half of the implant length. | ELISA | LIDS* | LIDS*** |
| Chaparro, Sanz (18) | CG: 17  DG: 18 | NI | NI | Absence of swelling, bleeding on probing, inflammation and suppuration; besides the absence of increased probing depth and the absence of radiographic bone loss | Presence of inflammation, as in peri-mucositis, but with the presence of progressive bone loss | Multiplex | HIDS* | HIDS |
| Chaparro, Beltran (12) | CG: 7  DG: 10 | 73.8 | 67.8 | Absence of visual signs of inflammation and bleeding on probing. | Presence of inflammation in the peri-implant mucosa and subsequent progressive loss of supporting bone. | Multiplex | HIDS | LIDS |
| Duarte, de Mendonça (13) | CG: 10 DG:15 | 49.1 | 55.8 | No marginal bleeding, BOP, suppuration and radiographic bone loss. | Implants with PD deeper or equal to 5mm, BOP and/or suppuration, radiographic bone loss involving at least three threads of the implant but no more than half of the implant length. | ELISA | HIDS | LIDS* |
| Milinkovic, Djinic Krasavcevic (14) | CG:35  DG:45 | 41,57 | 45,98 | Absence of clinical signs of inflammation; absence of bleeding and/or suppuration on gentle probing; no increase in PD; absence of bone loss beyond crestal bone level changes resulting  from initial bone remodeling | Presence of bleeding and/or suppuration on gentle probing; PD of ≥6 mm; bone levels ≥3 mm apical of the most coronal portion of the intraosseous part of the implant. | ELISA | HIDS | LIDS |
| Rakic, Struillou (15) | CG: 58  DG: 52 | 54,66 | 57,39 | These controls were defined by PD≤3mm, no BOP, and no bone loss evidence by radiograph. | Cases were defined by the presence of PD ≥5mm, with positive BOP and recorded radiographic bone loss involving at least two threads compared with radiographs taken at the time of the prosthetic replacement. | ELISA | HIDS* | HIDS* |
| Rakic, Petkovic-Curcin (19) | CG:189  DG:180 | 49.4 | 53.2 | When there was no implant with signs of inflammation (no BOP), no presence of pockets (PD≤3 mm), and without radiographically evidenced bone loss | Presence of PD ≥5 mm, with positive bleeding on probing and recorded radiographic bone loss involving ≥2 threads compared to the radiograph taken at the time of prosthetic replacement. | ELISA | HIDS*** | HIDS*** |
| Rakic, Lekovic (20) | CG: 25  DG:23 | 36 | 48 | When there was no implant with signs of inflammation (no BOP), no presence of pockets (PD≤3 mm), and without radiographically evidenced bone loss | Presence  of PPD ≥ 5 mm, with positive BOP and recorded RXBL ≥ 2 threads compared to the radiograph taken at the time of prosthetic replacement. | ELISA | HIDS* | HIDS* |
| Rakic, Monje (16) | CG: 126  DG:69 | NI | 53.14 | Implants with negative BOP or BOP positive in 1/6 sites being considered the consequence of trauma, with PD<3mm and without evidence of radiological bone loss. | PD ≥5 mm, BOP>1 and radiological bone loss involving ≥2mm compared to the radiograph taken at the time of prosthetic loading. | ELISA | HIDS*** | HIDS*** |
| Yakar, Guncu (21) | CG:25  DG:27 | 50.64 | 55.85 | No sign of inflammation, no sites with less than 4 mm probing depth and no evident radiographic bone loss at a relevant implant. | Presence of at least one peri-implant site with PD of ≥6 mm accompanied by at least one of the other signs as radiographic bone loss purulent exudate or bleeding | ELISA | HIDS**** | HIDS* |

CG: Control Group; DG: Disease Group; NI: Not informed; RANKL: Receptor Activator of Nuclear Factor Kappa-B Ligand; OPG: Osteoprotegerin; PD: Probing Depth; BOP: Bleeding On Probing; HIDS: Higher In Diseased Subjects; LIDS: Lower In Diseased Subjects. *p<0.05; ***p<0.001; ****p<0.0001.

| **Table S14:** Qualitative analysis of studies which focused on: RANKL versus OPG in peri-implant crevicular fluid; Mucositis versus Peri-implantitis | | | | | | | | |
| --- | --- | --- | --- | --- | --- | --- | --- | --- |
| **RANKL versus OPG – Peri-implant Crevicular Fluid - Mucositis versus Peri-implantitis** | | | | | | | | |
|  | | Mean age (year) | | Diagnostic Criteria | |  | | |
| Author Year | Sample Size | M | PI | M | PI | Evaluation Method | RANKL  Levels | OPG  Levels |
| Arıkan, Buduneli (11) | M: 4 PI: 3 | 52.8 | 66.3 | Implants with BOP, no suppuration or radiographic evidence of bone loss and no PD deeper than 5mm. | Implants with PD>5mm, BOP, suppuration and radiographic evidence of crestal bone loss in at least one site. | ELISA | HIPS | HIPS |
| Chaparro, Sanz (18) | M: 19  PI: 18 | NI | NI | Inflammation of the peri-implant soft tissues, without bone loss, but with bleeding on probing, swelling, and suppuration in some cases | Presence of inflammation, as in peri-mucositis, but with the presence of progressive bone loss. | Multiplex | HIPS*** | LIPS |
| Chaparro, Beltran (12) | M: 2  PI: 10 | 61.8 | 67.8 | Bleeding on probing and visual signs of inflammation. | Presence of inflammation in the peri-implant mucosa and subsequent progressive loss of supporting bone. | Multiplex | HIPS | LIPS |
| Duarte, de Mendonça (13) | M: 10 implants  PI: 20 implants | 55.8 | 55.8 | Implants with marginal bleeding and/or BOP, and absence of radiographic bone loss and suppuration. | Implants with PD ≥5mm, BOP and/or suppuration, radiographic bone loss involving at least three threads of the implant but no more than half of the implant length. | ELISA | HIPS | LIPS |
| Milinkovic, Djinic Krasavcevic (14) | M:50  PI:45 | 55,22 | 45,98 | Bleeding and/or suppuration on gentle probing with or without  increased PD; absence of continuing bone loss as observed on a radiograph: absence  of loss beyond crestal bone level changes resulting from initial bone remodeling. | Presence of bleeding and/or suppuration on gentle probing; PD of ≥6 mm; bone levels ≥3 mm apical of the most coronal portion of the intraosseous part of the implant. | ELISA | HIPS | LIPS |
| Rakic, Struillou (15) | M: 54  PI: 52 | 51,83 | 57,39 | Cases were defined by the presence of peri-implant PD≥3mm, with positive BOP and absence of radiographic bone loss compared with the radiograph taken at the time of prosthetic replacement. | Cases were defined by the presence of PD ≥5mm, with positive BOP and recorded radiographic bone loss involving at least two threads compared with radiographs taken at the time of the prosthetic replacement. | ELISA | HIPS | HIPS |
| Rakic, Monje (16) | M: 57 implants  PI: 69 implants | 52.5 | 53.14 | Implants with negative BOP or BOP positive in 1/6 sites being considered the consequence of trauma, with PD<3mm and without evidence of RXBL. | PD ≥5 mm, BOP>1 and RXBL involving ≥2mm compared to the radiograph taken at the time of prosthetic loading. | ELISA | LIPS | LIPS |

M: Mucositis; PI: Peri-implantitis; NI: Not informed; RANKL: Receptor Activator of Nuclear Factor Kappa-B Ligand; OPG: Osteoprotegerin; PD: Probing Depth; BOP: Bleeding On Probing; RXBL: Radiological bone loss; HIPS: Higher In Peri-implantitis Subjects; LIPS: Lower In Peri-implantitis Subjects. ***p<0.001.

| **Table S15:** Qualitative analysis of studies which focused on: RANKL versus OPG in tissue sample; Control versus Mucositis | | | | | | | | |
| --- | --- | --- | --- | --- | --- | --- | --- | --- |
| **RANKL versus OPG – Tissue sample - Control versus Mucositis** | | | | | | | | |
|  | | Mean age (year) | | Diagnostic Criteria | |  | | |
| Author Year | Sample Size | Control | Disease | Control | Disease | Evaluation Method | RANKL  Levels | OPG  Levels |
| Duarte, De Mendonça (22) | CG: 11  DG: 15 | 49.1 | 55.8 | Implants with PD≤4mm, without marginal bleeding, BOP, suppuration and radiographic evidence of bone loss. | Implants with marginal bleeding and/or BOP, absence of radiographic bone loss and suppuration. | PCR-RT | HIDS | LIDS* |

CG: Control Group; DG: Disease Group; RANKL: Receptor Activator of Nuclear Factor Kappa-B Ligand; OPG: Osteoprotegerin; PD: Probing Depth; BOP: Bleeding On Probing; HIDS: Higher In Disease Subjects; LIDS: Lower In Disease Subjects; *p<0.05.

| **Table S16:** Qualitative analysis of studies which focused on: RANKL versus OPG in tissue sample; Control versus Peri-implantitis | | | | | | | | | |
| --- | --- | --- | --- | --- | --- | --- | --- | --- | --- |
| **RANKL versus OPG – Tissue sample - Control versus Peri-implantitis** | | | | | | | | | |
|  | | Mean age (year) | | Diagnostic Criteria | |  | | | |
| Author Year | Sample Size | Control | Disease | Control | Disease | Evaluation Method | RANKL  Levels | OPG  Levels |  |
| Duarte, De Mendonça (22) | CG: 11  DG: 10 | 49.1 | 56.7 | Implants with PD ≤4mm, without marginal bleeding, BOP, suppuration and radiographic evidence of bone loss. | Implants with PD ≥5mm, with BOP and/or suppuration and radiographic bone loss involving four threads. | PCR-RT | HIDS* | LIDS* |  |
| Ghighi, Llorens (5) | CG:10  DG:11 | NI | NI | Patients underwent surgery for erupted third molar extraction and should not have either history of periodontitis or peri-implantitis according to clinical criteria of gingival bleeding, PD and radiographic evidence of bone resorptions. | Patients should present at least one dental implant in function with a titanium abutment, a PD≥5mm with BOP and radiographic evidence of bone loss. | Multiplex | HIDS*** | LIDS* |  |

CG: Control Group; DG: Disease Group; RANKL: Receptor Activator of Nuclear Factor Kappa-B Ligand; OPG: Osteoprotegerin; PD: Probing Depth; BOP: Bleeding On Probing; NI: Not informed; HIDS: Higher In Disease Subjects; LIDS: Lower In Disease Subjects. *p<0.05; ***p<0.001.

| **Table S17:** Qualitative analysis of studies which focused on: RANKL versus OPG in tissue sample; Control versus Peri-implantitis Severe | | | | | | | | |
| --- | --- | --- | --- | --- | --- | --- | --- | --- |
| **RANKL versus OPG – Tissue sample - Control versus Peri-implantitis Severe** | | | | | | | | |
|  | | Mean age (year) | | Diagnostic Criteria | |  | | |
| Author Year | Sample Size | Control | Disease | Control | Disease | Evaluation Method | RANKL  Levels | OPG  Levels |
| Duarte, De Mendonça (22) | CG: 11  DG: 12 | 49.1 | 59.4 | Implants with PD ≤4mm, without marginal bleeding, BOP, suppuration and radiographic evidence of bone loss. | Implants with PD ≥5mm with BOP and/or suppuration and radiographic bone loss involving more than four threads. | PCR-RT | HIDS* | LIDS* |

CG: Control Group; DG: Disease Group; RANKL: Receptor Activator of Nuclear Factor Kappa-B Ligand; OPG: Osteoprotegerin; PD: Probing Depth; BOP: Bleeding On Probing; HIDS: Higher In Disease Subjects; LIDS: Lower In Disease Subjects; *p<0.05.

| **Table S18:** Qualitative analysis of studies which focused on: RANKL versus OPG in tissue sample; Mucositis versus Peri-implantitis | | | | | | | | |
| --- | --- | --- | --- | --- | --- | --- | --- | --- |
| **RANKL versus OPG – Tissue sample - Mucositis versus Peri-implantitis** | | | | | | | | |
|  | | Mean age (year) | | Diagnostic Criteria | |  | | |
| Author Year | Sample Size | M | PI | M | PI | Evaluation Method | RANKL  Levels | OPG  Levels |
| Duarte, De Mendonça (22) | M: 15 PI: 10 | 55.8 | 56.7 | Implants with marginal bleeding and/or BOP, absence of radiographic bone loss and suppuration. | Implants with PD ≥5mm, with BOP and/or suppuration and radiographic bone loss involving four threads. | PCR-RT | HIPS* | HIPS* |

M: Mucositis; PI: Peri-implantitis; PD: Probing Depth; BOP: Bleeding On Probing; HIPS: Higher In Peri-implantitis Subjects; *p<0,05.

| **Table S19:** Qualitative analysis of studies which focused on: RANKL versus OPG in tissue sample; Mucositis versus Peri-implantitis Severe | | | | | | | | |
| --- | --- | --- | --- | --- | --- | --- | --- | --- |
| **RANKL versus OPG – Tissue sample - Mucositis versus Peri-implantitis Severe** | | | | | | | | |
|  | | Mean age (year) | | Diagnostic Criteria | |  | | |
| Author Year | Sample Size | M | PI | M | PI | Evaluation Method | RANKL  Levels | OPG  Levels |
| Duarte, De Mendonça (22) | M: 15 PI: 12 | 55.8 | 59.4 | Implants with marginal bleeding and/or BOP, absence of radiographic bone loss and suppuration. | Implants with PD ≥5mm, with BOP and/or suppuration and radiographic bone loss involving more than four threads. | PCR-RT | HIPS* | HIPS* |

M: Mucositis; PI: Peri-implantitis; PD: Probing Depth; BOP: Bleeding On Probing; HIPS: Higher In Peri-implantitis Subjects; *p<0,05.

**REFERENCES**

1. Casado PL, Canullo L, de Almeida Filardy A, Granjeiro JM, Barboza EP, Duarte MEL. Interleukins 1β and 10 expressions in the periimplant crevicular fluid from patients with untreated periimplant disease. Implant dentistry. 2013;22(2):143-50.

2. Guncu GN, Akman AC, Gunday S, Yamalik N, Berker E. Effect of inflammation on cytokine levels and bone remodelling markers in peri-implant sulcus fluid: a preliminary report. Cytokine. 2012;59(2):313-6.

3. Kandaswamy E, Sakulpaptong W, Guo X, Ni A, Powell HM, Tatakis DN, et al. Titanium as a Possible Modifier of Inflammation Around Dental Implants. Int J Oral Maxillofac Implants. 2022;37(2):381-90.

4. Ata-Ali J, Flichy-Fernandez AJ, Alegre-Domingo T, Ata-Ali F, Palacio J, Penarrocha-Diago M. Clinical, microbiological, and immunological aspects of healthy versus peri-implantitis tissue in full arch reconstruction patients: a prospective cross-sectional study. BMC oral health. 2015;15:43.

5. Ghighi M, Llorens A, Baroukh B, Chaussain C, Bouchard P, Gosset M. Differences between inflammatory and catabolic mediators of peri-implantitis and periodontitis lesions following initial mechanical therapy: An exploratory study. Journal of periodontal research. 2018;53(1):29-39.

6. Fonseca FJ, Moraes Junior M, Lourenco EJ, Teles Dde M, Figueredo CM. Cytokines expression in saliva and peri-implant crevicular fluid of patients with peri-implant disease. Clinical oral implants research. 2014;25(2):e68-72.

7. Teixeira MKS, Lira‐Junior R, Telles DM, Lourenço EJV, Figueredo CM. Th17‐related cytokines in mucositis: is there any difference between peri‐implantitis and periodontitis patients? Clinical oral implants research. 2017;28(7):816-22.

8. Song L, Jiang J, Li J, Zhou C, Chen Y, Lu H, et al. The Characteristics of Microbiome and Cytokines in Healthy Implants and Peri-Implantitis of the Same Individuals. J Clin Med. 2022;11(19).

9. Severino VO, Beghini M, de Araújo MF, de Melo MLR, Miguel CB, Rodrigues WF, et al. Expression of IL-6, IL-10, IL-17 and IL-33 in the peri-implant crevicular fluid of patients with peri-implant mucositis and peri-implantitis. Archives of oral biology. 2016;72:194-9.

10. Severino VO, Napimoga MH, de Lima Pereira SA. Expression of IL-6, IL-10, IL-17 and IL-8 in the peri-implant crevicular fluid of patients with peri-implantitis. Archives of oral biology. 2011;56(8):823-8.

11. Arıkan F, Buduneli N, Kütükçüler N. Osteoprotegerin levels in peri‐implant crevicular fluid. Clinical Oral Implants Research. 2008;19(3):283-8.

12. Chaparro A, Beltran V, Betancur D, Sam YH, Moaven H, Tarjomani A, et al. Molecular Biomarkers in Peri-Implant Health and Disease: A Cross-Sectional Pilot Study. Int J Mol Sci. 2022;23(17).

13. Duarte PM, de Mendonça AC, Máximo MBB, Santos VR, Bastos MF, Nociti Jr FH. Effect of anti‐infective mechanical therapy on clinical parameters and cytokine levels in human peri‐implant diseases. Journal of periodontology. 2009;80(2):234-43.

14. Milinkovic I, Djinic Krasavcevic A, Nikolic N, Aleksic Z, Carkic J, Jezdic M, et al. Notch down-regulation and inflammatory cytokines and RANKL overexpression involvement in peri-implant mucositis and peri-implantitis: A cross-sectional study. Clinical oral implants research. 2021;32(12):1496-505.

15. Rakic M, Struillou X, Petkovic-Curcin A, Matic S, Canullo L, Sanz M, et al. Estimation of bone loss biomarkers as a diagnostic tool for peri-implantitis. Journal of periodontology. 2014;85(11):1566-74.

16. Rakic M, Monje A, Radovanovic S, Petkovic‐Curcin A, Vojvodic D, Tatic Z. Is the personalized approach the key to improve clinical diagnosis of peri‐implant conditions? The role of bone markers. Journal of periodontology. 2020;91(7):859-69.

17. Arikan F, Buduneli N, Lappin DF. C-telopeptide pyridinoline crosslinks of type I collagen, soluble RANKL, and osteoprotegerin levels in crevicular fluid of dental implants with peri-implantitis: a case-control study. The International journal of oral & maxillofacial implants. 2011;26(2):282-9.

18. Chaparro A, Sanz A, Wolnitzky A, Realini O, Bendek MJ, Betancur D, et al. Lymphocyte B and Th17 chemotactic cytokine levels in peri-implant crevicular fluid of patients with healthy, peri-mucositis, and peri-implantitis implants. Journal of Oral Research. 2020:20-5.

19. Rakic M, Petkovic-Curcin A, Struillou X, Matic S, Stamatovic N, Vojvodic D. CD14 and TNFα single nucleotide polymorphisms are candidates for genetic biomarkers of peri-implantitis. Clinical oral investigations. 2015;19(4):791-801.

20. Rakic M, Lekovic V, Nikolic-Jakoba N, Vojvodic D, Petkovic-Curcin A, Sanz M. Bone loss biomarkers associated with peri-implantitis. A cross-sectional study. Clinical oral implants research. 2013;24(10):1110-6.

21. Yakar N, Guncu GN, Akman AC, Pınar A, Karabulut E, Nohutcu RM. Evaluation of gingival crevicular fluid and peri-implant crevicular fluid levels of sclerostin, TWEAK, RANKL and OPG. Cytokine. 2019;113:433-9.

22. Duarte PM, De Mendonça AC, Máximo MBB, Santos VR, Bastos MF, Nociti Júnior FH. Differential cytokine expressions affect the severity of peri‐implant disease. Clinical oral implants research. 2009;20(5):514-20.
