# Supplementary material for: Pro- and anti-inflammatory cytokines and osteoclastogenesis-related factors in peri-implant diseases: systematic review and meta-analysis
Source: BMC Oral Health. 2023 Jun 24;23:420. doi: 10.1186/s12903-023-03072-1 (PMC10290807; doi:10.1186/s12903-023-03072-1)
Supplement: Supplementary file 4 — Additional file 4: Table S20. Datas funding and limitations studies. [file 12903_2023_3072_MOESM4_ESM.docx]

**Table S20**: Datas funding and limitations studies

| **Author Year** | **Funding Data** | **Study Limitations** |
| --- | --- | --- |
| Arıkan, Buduneli (1) | Research Fund, Ege University, Turkey | The unbalanced sample in three health categories prevented from commenting on the possible role of sRANKL and OPG in bone loss around dental implants. |
| Arikan, Buduneli (2) | NI | The cross-sectional character is a possible limitation in the interpretation of the findings. |
| Ata-Ali, Flichy-Fernandez (3) | NI | Limited sample size. |
| Casado, Canullo (4) | NI | NI |
| Chaparro, Sanz (5) | Proyecto REDI170658, CONICYT-Chile and the Innovation Contest "Inicia Tu Idea Universidad de La Frontera-Universidad de Los Andes” | Limited sample size. |
| Chaparro, Beltran (6) | National Agency for Research and Development of Chile (ANID), National Institute for Health and Research of the United Kingdom | Limited sample size. |
| Duarte, de Mendonça (7) | FAPESP and CNPq | Limited sample size. |
| Duarte, De Mendonça (8) | FAPESP | Limited sample size. |
| Fonseca, Moraes Junior (9) | NI | Limited sample size and a large number of cytokines analyzed. |
| Ghighi, Llorens (10) | Dentsply IH, European Division Company | No clinical data were available for analysis and limited sample size. |
| Guncu, Akman (11) | Hacettepe University Research Foundation | NI |
| Kandaswamy, Sakulpaptong (12) | Osseointegration Foundation | Despite the large sample size, significant variability in cytokine concentrations was noted among individuals with the same clinical diagnosis. Moreover, clinical diagnostic tools, such as obtaining accurate and repeatable probing depths, have major limitations around implant-supported dental restorations, mainly due to restorative contours. |
| Milinkovic, Djinic Krasavcevic (13) | Ministry of Education, Science and Technological Development of Republic of Serbia | Limited sample size. |
| Rakic, Lekovic (14) | Ministry of Education and Science, Republic of Serbia | Limited sample size. |
| Rakic, Struillou (15) | Ministry of Education and Science, Republic of Serbia, Belgrade, Serbia | Limited sample size. |
| Rakic, Petkovic-Curcin (16) | Ministry of Education and Science, Republic of Serbia | Limited sample size. |
| Rakic, Monje (17) | Ministry of Education and Science, Republic of Serbia | NI |
| Song, Jiang (18) | Postdoctoral Science Foundation of China, Key Research and Development Program of Science and Technology Department of Zhejiang Province and The Zhejiang Provincial Natural Science Foundation of China | Limited sample size. |
| Severino, Napimoga (19) | University of Uberaba, Brazil and Federal University of Triangulo Mineiro | NI |
| Severino, Beghini (20) | Graduate Program in Health Sciences of the Federal University of Triangulo Mineiro, Cefores of Federal University of Triangulo Mineiro (UFTM), CNPq and FAPEMIG | NI |
| Teixeira, Lira‐Junior (21) | FAPERJ | Limited sample size and the study design (cross-sectional nature). |
| Yakar, Guncu (22) | Hacettepe University Research Foundation | Limited sample size, the study design (cross-sectional nature) and the wide range of ages in groups. |

NI: Not informed; FAPESP: Fundação de Amparo à Pesquisa do Estado de São Paulo; CNPq: Conselho Nacional de Desenvolvimento Científico e Tecnológico; FAPEMIG: Fundação de Amparo à Pesquisa do Estado de Minas Gerais; FAPERJ: Fundação de Amparo à Pesquisa do Estado do Rio de Janeiro.

**REFERENCES TABLE S20**

1. Arıkan F, Buduneli N, Kütükçüler N. Osteoprotegerin levels in peri‐implant crevicular fluid. Clinical Oral Implants Research. 2008;19(3):283-8.

2. Arikan F, Buduneli N, Lappin DF. C-telopeptide pyridinoline crosslinks of type I collagen, soluble RANKL, and osteoprotegerin levels in crevicular fluid of dental implants with peri-implantitis: a case-control study. The International journal of oral & maxillofacial implants. 2011;26(2):282-9.

3. Ata-Ali J, Flichy-Fernandez AJ, Alegre-Domingo T, Ata-Ali F, Palacio J, Penarrocha-Diago M. Clinical, microbiological, and immunological aspects of healthy versus peri-implantitis tissue in full arch reconstruction patients: a prospective cross-sectional study. BMC oral health. 2015;15:43.

4. Casado PL, Canullo L, de Almeida Filardy A, Granjeiro JM, Barboza EP, Duarte MEL. Interleukins 1β and 10 expressions in the periimplant crevicular fluid from patients with untreated periimplant disease. Implant dentistry. 2013;22(2):143-50.

5. Chaparro A, Sanz A, Wolnitzky A, Realini O, Bendek MJ, Betancur D, et al. Lymphocyte B and Th17 chemotactic cytokine levels in peri-implant crevicular fluid of patients with healthy, peri-mucositis, and peri-implantitis implants. Journal of Oral Research. 2020:20-5.

6. Chaparro A, Beltran V, Betancur D, Sam YH, Moaven H, Tarjomani A, et al. Molecular Biomarkers in Peri-Implant Health and Disease: A Cross-Sectional Pilot Study. Int J Mol Sci. 2022;23(17).

7. Duarte PM, de Mendonça AC, Máximo MBB, Santos VR, Bastos MF, Nociti Jr FH. Effect of anti‐infective mechanical therapy on clinical parameters and cytokine levels in human peri‐implant diseases. Journal of periodontology. 2009;80(2):234-43.

8. Duarte PM, De Mendonça AC, Máximo MBB, Santos VR, Bastos MF, Nociti Júnior FH. Differential cytokine expressions affect the severity of peri‐implant disease. Clinical oral implants research. 2009;20(5):514-20.

9. Fonseca FJ, Moraes Junior M, Lourenco EJ, Teles Dde M, Figueredo CM. Cytokines expression in saliva and peri-implant crevicular fluid of patients with peri-implant disease. Clinical oral implants research. 2014;25(2):e68-72.

10. Ghighi M, Llorens A, Baroukh B, Chaussain C, Bouchard P, Gosset M. Differences between inflammatory and catabolic mediators of peri-implantitis and periodontitis lesions following initial mechanical therapy: An exploratory study. Journal of periodontal research. 2018;53(1):29-39.

11. Guncu GN, Akman AC, Gunday S, Yamalik N, Berker E. Effect of inflammation on cytokine levels and bone remodelling markers in peri-implant sulcus fluid: a preliminary report. Cytokine. 2012;59(2):313-6.

12. Kandaswamy E, Sakulpaptong W, Guo X, Ni A, Powell HM, Tatakis DN, et al. Titanium as a Possible Modifier of Inflammation Around Dental Implants. Int J Oral Maxillofac Implants. 2022;37(2):381-90.

13. Milinkovic I, Djinic Krasavcevic A, Nikolic N, Aleksic Z, Carkic J, Jezdic M, et al. Notch down-regulation and inflammatory cytokines and RANKL overexpression involvement in peri-implant mucositis and peri-implantitis: A cross-sectional study. Clinical oral implants research. 2021;32(12):1496-505.

14. Rakic M, Lekovic V, Nikolic-Jakoba N, Vojvodic D, Petkovic-Curcin A, Sanz M. Bone loss biomarkers associated with peri-implantitis. A cross-sectional study. Clinical oral implants research. 2013;24(10):1110-6.

15. Rakic M, Struillou X, Petkovic-Curcin A, Matic S, Canullo L, Sanz M, et al. Estimation of bone loss biomarkers as a diagnostic tool for peri-implantitis. Journal of periodontology. 2014;85(11):1566-74.

16. Rakic M, Petkovic-Curcin A, Struillou X, Matic S, Stamatovic N, Vojvodic D. CD14 and TNFα single nucleotide polymorphisms are candidates for genetic biomarkers of peri-implantitis. Clinical oral investigations. 2015;19(4):791-801.

17. Rakic M, Monje A, Radovanovic S, Petkovic‐Curcin A, Vojvodic D, Tatic Z. Is the personalized approach the key to improve clinical diagnosis of peri‐implant conditions? The role of bone markers. Journal of periodontology. 2020;91(7):859-69.

18. Song L, Jiang J, Li J, Zhou C, Chen Y, Lu H, et al. The Characteristics of Microbiome and Cytokines in Healthy Implants and Peri-Implantitis of the Same Individuals. J Clin Med. 2022;11(19).

19. Severino VO, Napimoga MH, de Lima Pereira SA. Expression of IL-6, IL-10, IL-17 and IL-8 in the peri-implant crevicular fluid of patients with peri-implantitis. Archives of oral biology. 2011;56(8):823-8.

20. Severino VO, Beghini M, de Araújo MF, de Melo MLR, Miguel CB, Rodrigues WF, et al. Expression of IL-6, IL-10, IL-17 and IL-33 in the peri-implant crevicular fluid of patients with peri-implant mucositis and peri-implantitis. Archives of oral biology. 2016;72:194-9.

21. Teixeira MKS, Lira‐Junior R, Telles DM, Lourenço EJV, Figueredo CM. Th17‐related cytokines in mucositis: is there any difference between peri‐implantitis and periodontitis patients? Clinical oral implants research. 2017;28(7):816-22.

22. Yakar N, Guncu GN, Akman AC, Pınar A, Karabulut E, Nohutcu RM. Evaluation of gingival crevicular fluid and peri-implant crevicular fluid levels of sclerostin, TWEAK, RANKL and OPG. Cytokine. 2019;113:433-9.
